# De novo transcriptome analysis and functional annotation of *Silybum Marianum* L. under drought stress with a focus on Silymarin synthesis and MAPK signaling pathways

**DOI:** 10.1186/s12870-025-07272-5

**Published:** 2025-08-28

**Authors:** Rahele Ghanbari Moheb Seraj, Asadollah Ahmadikhah, Keyvan Esmaeilzadeh-Salestani, Vahid Shariati, Mahdi Behnamian, Neda Tariverdizadeh, Ali Emadi, Sara Dezhsetan

**Affiliations:** 1 Department of Horticultural Sciences, Faculty of Agriculture and Natural Resources, University of Mohaghegh Ardabili, Ardabil, Iran; 2 Department of Cell & Molecular Biology, Faculty of Life Sciences & Biotechnology, Shahid Beheshti University, Tehran, Iran; 3 Chair of Crop Science and Plant Biology, Institute of Agricultural and Environmental Sciences, Estonian University of Life Sciences, Kreutzwaldi 1, Tartu, 51006 Estonia; 4 Institute of Technology, University of Tartu, Nooruse 1, Tartu, 50411 Estonia; 5 Department of Plant Molecular Biotechnology, National Institute of Genetic Engineering and Biotechnology, Tehran, Iran; 6 Department of Biotechnology, Iranian Research Organization for Science and Technology (IROST), Tehran, Iran; 7 Department of Agriculture and Plant Breeding group, Faculty of Agriculture and Natural Resources, University of Mohaghegh Ardabili, Ardabil, Iran

**Keywords:** RNA-Seq, Silybinin, Water shortage, Gene ontology, KEGG pathway, MAPK.

## Abstract

To evaluate the milk thistle transcriptome under drought stress in field conditions, irrigation was applied using a weighted method at three levels: 100% F.C every 2 days, 70% F.C every 4 days, and 40% F.C every 8 days. Sampling was performed after 8 days at the flowering stage. Plant leaves were collected for RNA-seq analysis, seeds for oily and methanolic extracts, and downstream analyses were performed. Since there was no annotated reference genome for this plant, the De novo Assembly method was implemented to assemble the transcriptome. Contigs were blasted against five databases: NT, NR, Uniprot, and protein databases of *Arabidopsis thaliana* and *Helianthus annuus*. A total of 9,517 genes (~ 73% of Uniprot genes) were common across all databases and selected for further analysis due to their comprehensive annotation. Then, DEGs were identified and functionally annotated using Gene Ontology (GO) analysis with the ShinyGO platform, biological pathway analysis through KEGG, and transcription factor identification via PlantTFDB. Next, silybinin content was measured using HPLC. Generally, the most repeated pathways in all treatments include the Biosynthesis of secondary metabolites and the MAPK signaling pathway. Also, most biological processes are related to the oxidation-reduction process, and response to stress, and most molecular functions are protein and mRNA binding. Our results indicate the active role of transcription factors ERF, C3H, and bHLH in drought stress tolerance. Silybin a and b showed that severe drought stress enhanced the accumulation of silybinin compared with seeds from the control. Eight differentially expressed genes (CYP86A1, CYP710A1, FATA2, LACS3, LOX2, PAL, PLA2-ALPHA, and PXG3) were used to validate the RNA-Seq data. qRT-PCR results confirmed strong consistency with the RNA-Seq findings. Finally, the genes involved in the silymarin pathway were identified, and their expression was determined through RNA-Seq data and compared with the silymarin contents.

**Supplementary Information:**

The online version contains supplementary material available at 10.1186/s12870-025-07272-5.

## Introduction

Milk thistle (*Silybum marianum* (L.) Gaertn) is an herbaceous plant, an annual or biennial, from the Asteraceae family, which has spread to Mediterranean climates [1]. This plant is commercially cultivated for medicinal purposes in dry rocky soils in Europe, China, Australia, Canada, and North and South America [2]. For more than 2000 years, the fruit of Milk thistle has served as a treatment for liver disorders [3]. The plant’s fruit contains silymarin, a biologically active compound consisting of flavonolignans such as silybin, isosilybin, taxifolin, silydianin, and silychristin [4]. Silybin, comprising 60–70% of silymarin, is a key component with significant benefits [5]. This compound protects against a wide range of liver diseases, including toxic and viral hepatitis, cirrhosis, fatty liver, and damage to liver cells caused by various toxic substances [6]. Milk thistle has recently been characterized as having antioxidant, antidepressant, anticancer, cardioprotective, demulcent, digestive tonic, hepatoregenerative, hepatoprotective, immunostimulatory, and neuroprotective properties [7]. Therefore, as a result of the advantageous properties of the fruit of this medicinal plant, its cultivation is increasing on a global scale [1].

Plants are subjected to a range of environmental stressors, including temperature fluctuations, drought, salinity, and exposure to heavy metals, all of which have the potential to affect the growth and development of plants [8–10]. In arid and semi-arid regions, water scarcity is the primary limiting factor for plant yield [11]. Studying drought stress has gained significant importance due to the increasing global drought trend and water resource depletion. Additionally, the relative drought tolerance of milk thistle provides a valuable opportunity to investigate the molecular, phytochemical, and physiological mechanisms underlying plant adaptation to this stress. Drought is a physicochemical phenomenon that disrupts the structural integrity of biological molecules, encompassing proteins, nucleic acids, fatty acids, carbohydrates, hormones, nutrients, and ions [12]. Plant adaptation to drought involves intricate and systematic molecular mechanisms. These mechanisms encompass water channel proteins, stress-responsive proteins, transcription factors, and signaling pathways, all of which are pivotal constituents of the plant’s stress response [13].

Transcriptome sequencing via RNA-seq technology offers a rapid, cost-effective, and efficient approach for unraveling the functionality of novel genes. Furthermore, it provides valuable insights into gene expression and regulation, particularly in plant species lacking complete genome sequences [14, 15]. In the absence of a good reference genome, the de novo assembly of an organism’s transcriptome serves as a valuable technique to identify responsive genes or transcripts to different treatments. Additionally, it facilitates a comprehensive understanding of the expression profile revealed by the candidate genes [16]. In a publication by Fulvio et al. [17], it was noted that more than 300 studies focusing on the medicinal properties of *S. marianum* have been documented in the PubMed database. This underscores the current scientific interest in milk thistle within international herbal plant research communities.

Although numerous studies have been conducted on *S. marianum*, few have focused on molecular investigations such as genetic diversity and gene expression analysis. Therefore, molecular studies on this plant appear to be essential [18, 19]. So far, only two transcriptome-related projects for *Silybum marianum* exist in the NCBI database. Only one (Accession: PRJNA659420), associated with this study, has examined the plant’s transcriptomic response to drought stress. The milk thistle’s genome documentation in the NCBI database indicates that a genome annotation file in GFF format has not yet been generated. As the plant’s genome was not annotated to find and annotate differentially expressed genes (DEGs) and conduct enrichment analyses such as gene ontology (GO) and Kyoto Encyclopedia of Genes and Genomes (KEGG), we sequenced the RNAs, performed de novo assembly of the reads, and created a reference transcriptome.

The main objectives of this study are as follows: [1] To perform a comprehensive transcriptome analysis of S. marianum to identify genes involved in the drought stress response; [2] To functionally annotate differentially expressed genes (DEGs) to elucidate their roles in drought-related biological pathways; [3] To quantify key metabolites in S. marianum and analyze their biosynthetic pathways; [4] To identify genes involved in the biosynthetic pathways of the selected metabolites and analyze their expression patterns using transcriptomic data [5]. To compare metabolite variations with gene expression profiles, to understand the interplay between molecular regulation and phytochemical production under drought conditions.

## Results

### Evaluation and cleaning of reads

Utilizing FastQC for initial data quality assessment, adapter sequences and low-quality nucleotides were excised via the Trimmomatic tool, applying a SLIDINGWINDOW: 5:20 average quality criterion and a minimum sequence length of 50 bp (MINLEN: 50). As summarized in Table 1, the vast majority of read pairs (99.98%) were retained after trimming, with only a minimal fraction of reads removed or present as singletons, indicating a high-quality dataset suitable for downstream analyses. Quality re-assessment with FastQC confirmed the efficacy of the trimming process.

**Table 1:** Post-trimming statistics of sequencing reads following quality trimming

Read types	Read Counts	Read percent (%)
Survived read pairs	32,515,278	99.98
Single forward reads	7974	0.02
Single reverse reads	5	< 0.01
Removed read pairs	3	< 0.01
Total input pairs	32,523,260	100

### De Novo assembly and BLAST

High-quality, trimmed reads were subjected to assembly using the Trinity software, generating 159,295 contigs. These contigs underwent subsequent analysis through BLAST alignment against several databases, including the NT, NR, and Uniprot databases, as well as *Arabidopsis thaliana* and *Helianthus annuus* protein databases, to elucidate protein information. The analysis identified a total of 282,012 contigs in the NT database, 128,917 contigs in the NR database, 13,077 contigs in the Uniprot database, 73,744 contigs associated with *Arabidopsis thaliana*, and 117,508 contigs related to *Helianthus annuus* databases.

**Fig. 1:**
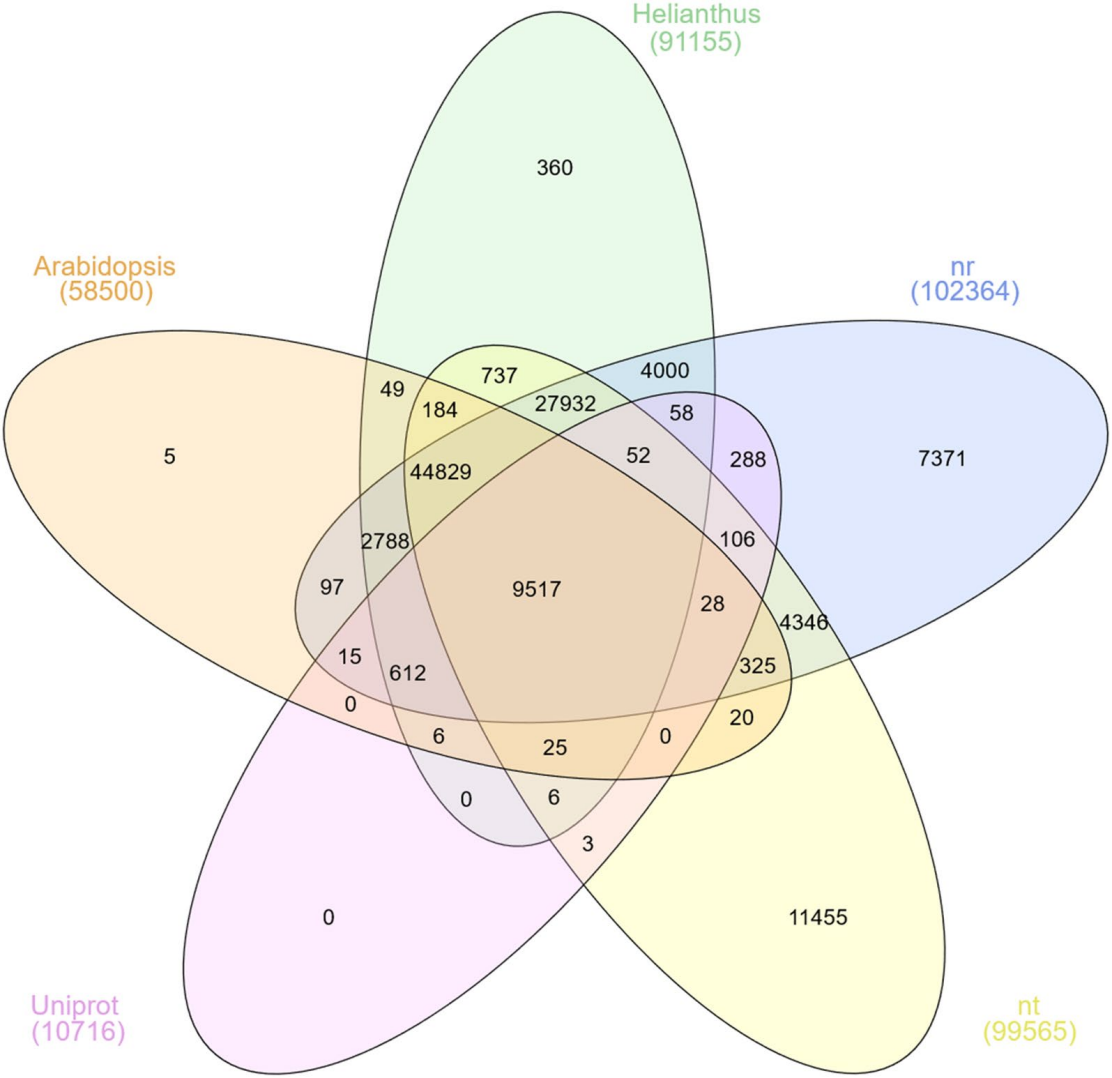
Venn diagram illustrating the comparison between genes in five databases

The comparative analysis revealed that 9,517 genes were commonly found across all five examined databases, representing approximately 73% of the total genes identified in the Uniprot database. Furthermore, about 5% of the genes listed in Uniprot were also present in the four other databases, while the remaining genes were commonly present between two or three databases. For subsequent downstream analyses, we focused on contigs identified through the Uniprot database. This decision was based on several considerations: (i) Uniprot IDs have broad compatibility and overlap with contigs annotated in other databases, (ii) many commonly used tools and databases recognize and support Uniprot identifiers, and (iii) Uniprot provides more comprehensive and well-curated annotations, facilitating more reliable functional interpretation and biological insights (Fig. 1).

### Gene ontology classification

A GO analysis was carried out using genes from Uniprot, concentrating on biological processes, molecular function, cellular components, and protein class. The study involved selecting and listing 10 significant GO terms for each category that had a high number of genes (Sup1). The predominant biological processes identified were metabolic and cellular processes, response to stimuli, and stress. The prevalent molecular functions included catalytic activities, binding, transferase, and hydrolase activities. Enriched cell components encompassed intracellular organelles, cytoplasm, chloroplasts, and membranes. Genes obtained from Uniprot were frequently classified into protein classes such as enzymes, transporters, and signal receptors.

### Identification of functional KEGG pathways

The pathways of Uniprot genes were identified and listed in sup2, including the number of genes involved in each pathway alongside KO identifications. An ensemble of 4,243 genes participates in 384 distinct pathways. There were two pathways of paramount importance in the milk thistle plant. The first pathway is the flavonoid biosynthesis pathway, which facilitates silymarin production. This pathway engages eight Uniprot genes. The second pathway is the biosynthesis pathway of unsaturated fatty acids, comprising linoleic acid, palmitic acid, oleic acid, etc. This pathway similarly includes eight Uniprot genes.

The protein families associated with these genes are systematically categorized into three groups (metabolism, genetic information processing, and signaling in juxtaposition with cellular processes) using the KEGG web server (Sup3). The metabolism category encompasses a diverse array of subgroups, including enzymes, protein phosphatases and associated proteins, protein kinases, glycosyltransferases, lipid and lipopolysaccharide biosynthesis proteins, peptidases and inhibitors, prenyltransferases, peptidoglycan biosynthesis and degradation proteins, cytochrome p450, amino acid related enzymes, and photosynthesis proteins.

The genetic information processing group was constitutive of subcategories such as transcription factors, messenger RNA biogenesis, transcription machinery, spliceosome, translation factors, ribosome biogenesis, transfer RNA biogenesis, membrane trafficking, ubiquitin system, chaperones and folding catalysts, DNA replication proteins, proteasome, chromosome and associated proteins, mitochondrial biogenesis, and DNA repair and recombination proteins. The signaling and cellular process group included subgroups of transporters, secretion system, cilium and related proteins, exosome, cytoskeleton proteins, G protein-coupled receptors, prokaryotic defense system, pattern recognition receptors, ion channels, GTP-binding proteins, CD molecules, glycosylphosphatidylinositol (GPI)-anchored proteins, glycosaminoglycan binding proteins, and domain-containing proteins. Each subgroup underwent further subdivision, with specific gene names in the detailed components listed below. Notably, these genes corresponded with those indexed by Uniprot, underpinning their significance in the identified pathways and protein families.

### Identification of DEGs

In three different treatments (70% F.C vs. F.C, 40% F.C vs. F.C, and 40% F.C vs. 70% F.C), DEGs (both up-and down-regulated) were identified. The criteria for identifying these genes were − 2 ≤ Log Fold Change (Log FC) ≥ + 2 and P-Value ≤ 0.01. In the 70% F.C vs. F.C comparison, we found 4,593 up-regulated genes (Log FC ranging from + 2 to + 18) and 6,741 down-regulated genes (Log FC ranging from − 2 to −20). In the 40% F.C vs. F.C comparison, there were 3530 up-regulated genes (Log FC range: +2 to + 15) and 4793 down-regulated genes (Log FC range: −2 to −18). In the 40% F.C vs. 70% F.C comparison, we identified 5936 up-regulated genes (Log FC range: +2 to + 20) and 5732 down-regulated genes (Log FC range: −2 to −18).

### Comparison of gene expression in three irrigation treatments of 100%, 70% and 40% of field capacity

**Fig. 2:**
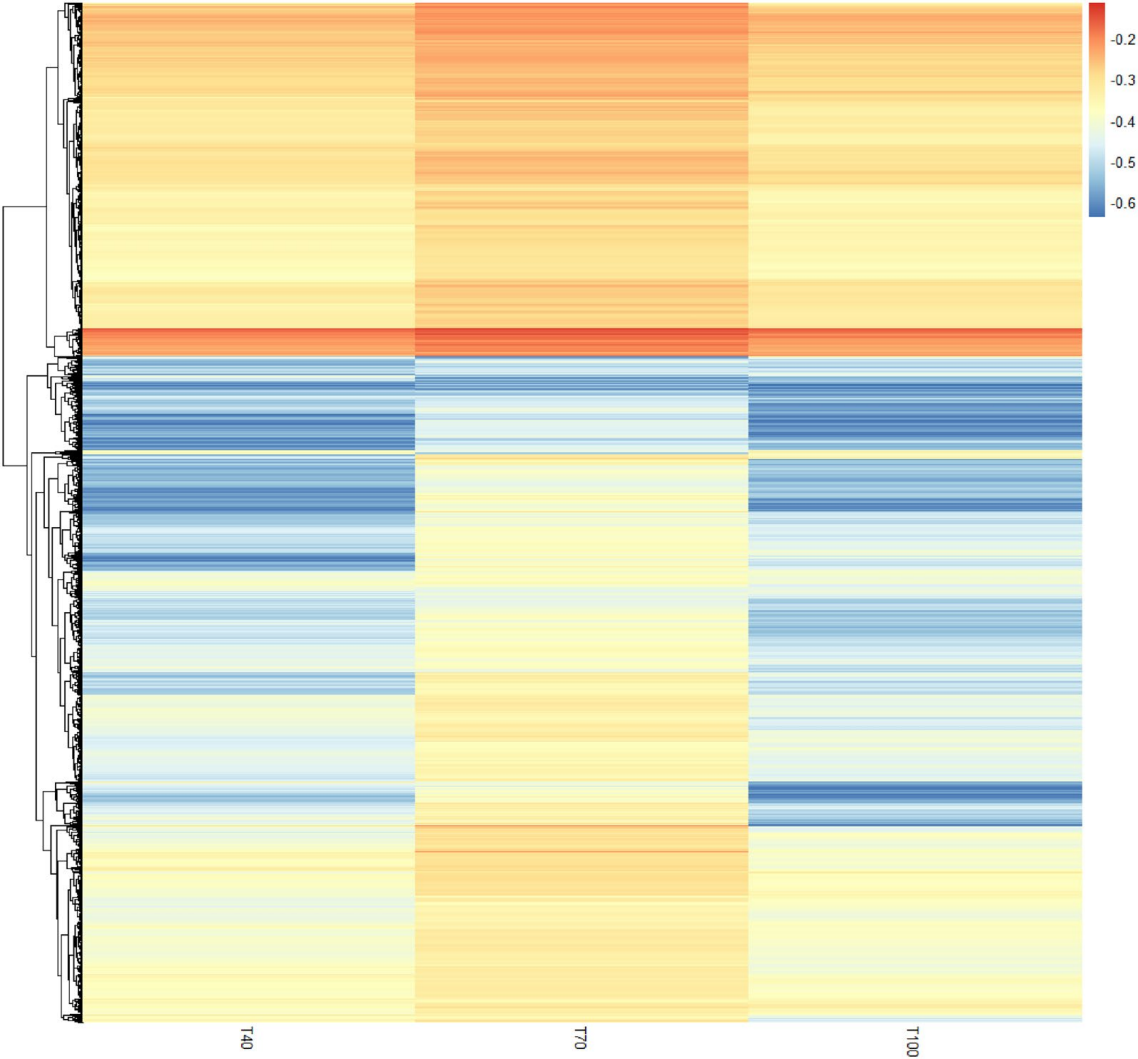
Comparison of gene expression in three irrigation treatments at 100, 70 and 40% of field capacity

An analytical comparison was conducted to assess the expression levels of 159,295 genes in three different treatments. The gene expression levels ranged from Log FC zero to 690,587. To facilitate a better comparison of gene expression in the three treatments, genes with expression levels of |Log2 FC| ≥ 2 were excluded. The analysis proceeded with a revised dataset comprising 21,151 genes. The comparative evaluation of gene expressions under three different degrees of drought stress was visualized employing a heatmap, wherein the gene expression data were subjected to Log10 FC to enhance clarity and interpretability. The analysis demonstrated that genes subjected to irrigation treatment at 70% of field capacity had a higher expression level than those under 100% and 40% field capacity treatments (Fig. 2), suggesting differential gene expression responses under varied irrigation regimes.

### Functional KEGG pathways identification of differential expression genes

Following the identification of up- and down-regulated genes in each pairwise comparison (70% F.C vs. F.C, 40% F.C vs. F.C, and 40% F.C vs. 70% F.C), the corresponding activity pathways for these gene groups were determined. In the 70% vs. F.C pairwise comparison, 123 different activity pathways were identified for up-regulated genes, with 29 pathways found to be significant at the ≤ 0.01 level. For down-regulated genes in the same comparison, 122 activity pathways were identified, with 30 pathways showing p-values ≤ 0.01. In the 40% vs. F.C pairwise comparison, 125 pathways were identified for up-regulated genes, with 28 pathways significant at the ≤ 0.01 level, while for down-regulated genes, 125 activity pathways were identified, with 26 pathways significant at the p-value of ≤ 0.01. In the 40% vs. 70% pairwise comparison, 124 different pathways were identified for up-regulated genes, with 26 pathways having a p-value of ≤ 0.01. For down-regulated genes in the same comparison, 124 activity pathways were identified, with 46 having a p-value ≤ 0.01. Subsequently, for each gene group, the 10 activity pathways with a p-value ≤ 0.01 and the highest number of genes were selected and listed in Table 2.

**Table 2:** KEGG pathway analysis of milk Thistle Up- and Down-regulated genes under three levels of water stress

Up-regulated genes in 70 vs 100% F.C treatments				Down-regulated genes in 70 vs 100% F.C treatments			
KEGG Pathway		GenesNo.	FDR adj.	KEGG Pathway	GenesNo.		FDRadj.
Metabolic pathways		544	1.70E-45	Metabolic pathways	681		9.06E-41
Biosynthesis of secondary metabolites		264	3.41E-20	Biosynthesis of secondary metabolites	315		7.39E-15
Carbon metabolism		77	1.97E-08	Carbon metabolism	79		3.91E-04
Biosynthesis of amino acids		70	1.39E-07	Biosynthesis of amino acids	67		5.95E-03
Ribosome		69	4.79E-03	Spliceosome	64		7.84E-05
Protein processing in endoplasmic reticulum		60	8.15E-07	Protein processing in endoplasmic reticulum	60		3.37E-03
MAPK signaling pathway		53	7.44E-10	Oxidative phosphorylation	54		3.04E-04
Starch and sucrose metabolism		49	4.36E-06	Starch and sucrose metabolism	53		8.21E-04
Spliceosome		47	4.59E-04	Glyoxylate and dicarboxylate metabolism	42		3.64E-07
Plant-pathogen interaction		42	8.88E-04	mRNA surveillance pathway	38		4.18E-03
**Up-regulated genes in 40 vs 100% F.C treatments**				**Down-regulated genes in 40 vs 100% F.C treatments**			
Metabolic pathways		358	1.05E-17	Metabolic pathways	493		6.23E-28
Biosynthesis of secondary metabolites		184	4.75E-10	Biosynthesis of secondary metabolites	251		4.44E-15
Carbon metabolism		54	1.76E-04	Carbon metabolism	101		5.47E-16
Biosynthesis of amino acids		43	6.81E-03	Biosynthesis of amino acids	73		8.16E-08
Starch and sucrose metabolism		32	6.09E-03	Spliceosome	58		9.59E-07
Spliceosome		32	2.99E-03	Protein processing in endoplasmic reticulum	56		4.51E-05
Glyoxylate and dicarboxylate metabolism		31	1.06E-04	Oxidative phosphorylation	42		7.15E-04
Glycolysis/Gluconeogenesis		21	6.52E-03	Glycolysis/Gluconeogenesis	39		1.77E-05
RNA degradation		20	9.60E-03	Glyoxylate and dicarboxylate metabolism	37		4.41E-08
Glycine, serine and threonine metabolism		19	3.49E-03	mRNA surveillance pathway	35		2.99E-04
**Up-regulated genes in 40 vs 70% F.C treatments**				**Down-regulated genes in 40 vs 70% F.C treatments**			
Metabolic pathways		624	1.22E-41	Metabolic pathways	685		1.42E-63
Biosynthesis of secondary metabolites		297	6.14E-17	Biosynthesis of secondary metabolites	347		1.65E-32
Carbon metabolism		68	1.98E-03	Ribosome	141		3.81E-19
Oxidative phosphorylation		55	1.10E-05	Carbon metabolism	131		4.40E-24
Starch and sucrose metabolism		54	4.19E-05	Biosynthesis of amino acids	114		1.79E-19
Glycolysis/Gluconeogenesis		40	2.36E-04	Protein processing in endoplasmic reticulum	103		2.57E-19
Ubiquitin mediated proteolysis		40	4.32E-04	RNA transport	66		4.63E-09
MAPK signaling pathway		38	4.75E-04	Glycolysis/Gluconeogenesis	62		5.12E-13
Glyoxylate and dicarboxylate metabolism		37	2.39E-06	Oxidative phosphorylation	59		1.27E-07
Fatty acid metabolism		27	4.78E-05	MAPK signaling pathway	54		5.22E-08

### Gene ontology classification of differential expression genes

This annotation systematically classified the DEGs into three principal categories based on their functional characteristics: biological process, molecular function, and cellular component. Six different gene groups were identified based on their expression profiles (up-regulated and down-regulated) across three pairwise comparisons of 70% F.C vs. F.C, 40% F.C vs. F.C, and 40% F.C vs. 70% F.C. The functions that were statistically significant (*P* ≤ 0.01) and encompassed the highest number of genes within these groups were presented in Table 3.

**Table 3:** Gene ontology analysis of milk Thistle Up- and Down-regulated genes under three levels of water stress

	Up-regulated genes in 70 vs. 100% F.C treatments			Down-regulated genes in 70 vs. 100% F.C treatments		
	Gene Ontology	Genes No.	FDR adj.	Gene Ontology	Genes No.	FDR adj.
Biological process	Protein phosphorylation	181	4.14E-13	Protein phosphorylation	250	8.77E-17
	Response to cadmium ion	144	4.23E-34	Oxidation-reduction process	149	2.48E-08
	Response to abscisic acid	112	2.02E-13	Embryo development ending in seed dormancy	145	2.07E-10
	Response to cold	111	6.53E-18	Response to abscisic acid	139	1.36E-12
	Response to water deprivation	109	1.60E-19	Signal transduction	133	2.51E-10
Molecular function	Protein binding	766	7.35E-65	Protein binding	1013	2.35E-71
	mRNA binding	308	1.85E-51	mRNA binding	288	2.04E-21
	ATP binding	158	1.92E-12	ATP binding	242	3.93E-22
	Zinc ion binding	124	2.11E-08	Protein kinase activity	178	1.84E-17
	Protein kinase activity	123	7.50E-12	Zinc ion binding	153	6.25E-07
Cellular component	Nucleus	1429	3.30E-23	Nucleus	2032	1.28E-37
	Cytoplasm	895	7.35E-65	Chloroplast	1176	2.00E-52
	Chloroplast	894	6.03E-51	Cytoplasm	1060	1.01E-42
	Cytosol	876	5.70E-113	Cytosol	952	1.41E-65
	Plasma membrane	712	9.08E-60	Plasma membrane	881	8.82E-51
	**Up-regulated genes in 40 vs. 100% F.C treatments**			**Down-regulated genes in 40 vs. 100% F.C treatments**		
Biological process	Protein phosphorylation	163	3.84E-16	Protein phosphorylation	183	3.09E-12
	Oxidation-reduction process	87	1.39E-05	Embryo development ending in seed dormancy	123	2.74E-12
	Protein ubiquitination	84	2.39E-05	Oxidation-reduction process	115	1.89E-07
	Embryo development ending in seed dormancy	76	8.89E-05	Defense response to bacterium	103	7.90E-13
	Protein autophosphorylation	62	1.47E-14	Response to salt stress	93	9.89E-09
Molecular function	Protein binding	539	2.12E-33	Protein binding	759	2.40E-56
	mRNA binding	177	6.22E-17	mRNA binding	258	8.72E-30
	ATP binding	142	5.00E-15	ATP binding	203	1.60E-24
	Protein kinase activity	117	3.97E-16	RNA binding	132	5.63E-16
	Zinc ion binding	109	1.37E-09	Zinc ion binding	129	1.23E-08
Cellular component	Nucleus	1197	1.33E-29	Nucleus	1528	1.22E-30
	Chloroplast	639	2.35E-25	Cytoplasm	866	3.63E-50
	Cytoplasm	610	2.03E-27	Chloroplast	865	7.38E-38
	Cytosol	527	1.87E-35	Cytosol	798	4.99E-77
	Plasma membrane	519	2.49E-35	Plasma membrane	654	2.09E-38
	**Up-regulated genes in 40 vs. 70% F.C treatments**			**Down-regulated genes in 40 vs. 70% F.C treatments**		
Biological process	Protein phosphorylation	228	1.19E-16	Response to cadmium ion	201	3.60E-54
	Oxidation-reduction process	133	1.39E-07	Protein phosphorylation	182	5.94E-08
	Embryo development ending in seed dormancy	118	7.14E-07	Oxidation-reduction process	143	4.12E-11
	Response to abscisic acid	105	1.26E-06	Embryo development ending in seed dormancy	137	9.72E-13
	Rrotein ubiquitination	106	2.03E-03	Response to water deprivation	121	3.81E-19
Molecular function	Protein binding	839	4.81E-49	mRNA binding	404	6.93E-75
	mRNA binding	270	2.99E-23	ATP binding	188	2.24E-14
	ATP binding	199	1.04E-15	RNA binding	153	1.53E-18
	Protein kinase activity	161	7.79E-19	Protein kinase activity	114	7.07E-06
	Protein serine/threonine kinase activity	144	6.09E-21	Structural constituent of ribosome	105	5.62E-15
Cellular component	Nucleus	1808	2.29E-34	Nucleus	1668	3.33E-22
	Chloroplast	1040	2.00E-46	Cytosol	1168	1.31E-180
	Cytoplasm	963	4.30E-43	Cytoplasm	1150	1.15E-99
	Cytosol	823	6.02E-53	Chloroplast	1022	6.51E-49
	Plasma membrane	800	6.42E-50	Mitochondrion	663	3.34E-04

### Metabolic pathway analysis

**Fig. 3:**
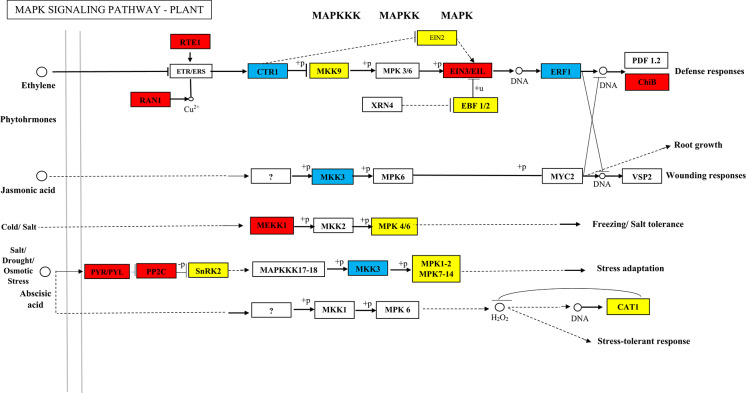
MAPK signaling pathway map of Milk thistle up- and down-regulated genes under drought stress condition. The up-regulated genes are in red, the down-regulated genes are in blue, and the genes that were in both up- and down-regulated gene categories are in yellow

The analysis linking significant DEGs with KEGG pathways identified the MAPK signaling pathway as significantly implicated (Fig. 3). This pathway began when cell membranes detected drought-related signals, subsequently regulated by PRY/PRL as soluble receptors for abscisic acid (ABA) in an ABA-dependent mechanism. The 2 C-type protein phosphatases (PP2C), responsible for the deactivation of SNF1-related protein kinases 2 (SnRK2) through dephosphorylation, were suppressed in the presence of ABA. This investigation revealed a significant upregulation in the PRY/PRL and PP2C gene expressions. The activation of SnRK2 plays a pivotal role against biotic stressors such as salinity and drought by stimulating plant defensive responses and modulating transcriptional factors to increase the biosynthesis of functional proteins. The SnRK2 gene showed both up- and downregulation in our study.

The MAPK signaling cascade followed a classical pattern where MAPKKK was activated by plasma membrane receptors, which subsequently transmitted signals downstream. This led to the activation of MAPKK through the phosphorylation of the S/T-XXXXX-S/T motif, followed by MAPKK catalyzing the phosphorylation of the TXY motif in MAPK. Ultimately, MAPK activated a series of downstream enzymes, kinases, transcription factors, and additional response elements, translating extracellular environmental signals into cellular responses. However, our data indicated a reduction in MKK3 expression, whereas MPK7/14 expression varied, showing both increases and decreases.

**Fig. 4:**
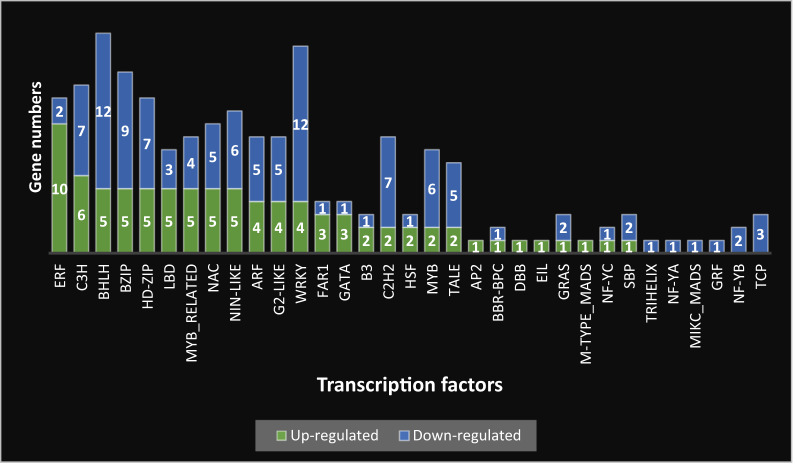
Transcription factors identified in milk thistle transcriptome under water stress treatment. The green boxes correspond to up-regulated genes, and the blue boxes correspond to down-regulated genes. The numbers inside the boxes represent the number of genes

Additionally, our findings underscored the critical role of ethylene, a hormone significantly influenced under drought stress conditions (Fig. 4). Ethylene interaction with its receptors was mediated by a copper co-factor, presumably supplied by the copper transporters RAN1 and RTE1, which were found to be upregulated in our study—this interaction inactivated receptor functionality. The histidine kinase ETR1/ERS1 (an ethylene receptor) activated CTR1 in ethylene’s absence. CTR1 acted as a negative regulator of the MAPKK SIMKK and MPK3/6 in Milk thistle, with its inactivation upon ethylene’s presence, thereby relieving SIMKK from inhibition and allowing for the activation of MAPKs. This cascade stimulated the expression of ethylene-responsive genes through direct activation of EIN2 and EIN3 or other intermediaries.

### Transcription factors identification

In the study of Milk thistle subjected to drought conditions, a comprehensive analysis of its transcriptome revealed the identification of 33 different transcription factors. These transcription factors and their expressions, quantified as either up-regulated or down-regulated, were cataloged in Fig. 4. The ERF transcription factor category showed the most substantial increase in expression, with 10 factors being up-regulated, closely followed by the C3H with six up-regulated factors. Transcription factors BHLH, BZIP, HD-ZIP, LBD, MYB_RELATED, NAC, and NIN-LIKE demonstrated comparable levels of expression change. Among down-regulated transcription factors, the BHLH and WRKY categories showed reduced expression, each with 12 factors, followed by the BZIP category with nine down-regulated factors. The number of down-regulated transcription factors in the Milk thistle transcriptome was significantly higher than up-regulated factors. Among the transcription factors analyzed, several transcription factors, such as TRIHELIX, NF-YA, MIKC-MADS, GRF, NF-YB, and TCP, were exclusively found to be down-regulated, with no corresponding increase in any transcription factors within these categories.

Trihelix transcription factors, characterized by a unique trihelix (helix-loop-helix-loop-helix) structure, are plant-specific and bind to GT elements in gene promoters. They play key roles in light response, growth regulation, organ development, and stress responses. This family is divided into five subgroups, with structural variations contributing to their functional diversity. Trihelix genes are involved in processes such as embryonic development, floral morphogenesis, and stomatal regulation, highlighting their importance in plant physiological regulation [20, 21]. The downregulation of the Trihelix transcription factor in our experiment may be related to its role as a negative regulator in stress responses. Some members of this family, such as ASIL1, repress growth- or stress-related genes, and under drought conditions, the suppression of such factors could be part of the plant’s strategy to enhance stress tolerance [22].

On the other hand, MIKC-MADS transcription factors are a subgroup of MADS proteins in plants that regulate the growth and development of various organs such as roots, stems, flowers, and fruits. These factors bind to two specific sites on DNA by forming heterotetramers, causing DNA looping, which increases the accuracy of target gene recognition and plays a vital role in biological functions and agriculture [23]. The decreased expression of MIKC-MADS in your experiment is likely a response to drought stress. The plant reduces the expression of developmental transcription factors like MIKC-MADS to conserve energy, slow down growth, and redirect resources toward stress adaptation [24]. Together, the reduced expression of Trihelix and MIKC-MADS transcription factors underscores a coordinated regulatory adjustment by plants to optimize survival under adverse conditions.

### Expression of DEGs using qRT-PCR

An analysis was conducted to evaluate the differential expression of targeted genes, focusing on both up-regulated and down-regulated genes under different irrigation conditions. This study specifically examined the expression profiles of four up-regulated genes, including PXG3, LOX2, CYP710A1, and Palmitoyl-protein thioesterase (AT5G47330), and four down-regulated genes, including FATA2, CYP86A1, LACS3, and PLA2-ALPHA under three different irrigation levels (three replicates).

Our findings revealed that PXG3 expression significantly increased by 65.119 and 59.302-fold under 40% F.C and 70% F.C irrigation levels, respectively, compared to the F.C condition. Palmitoyl-protein thioesterase (PAL) significantly increased expression at 70% F.C compared to the control (154.879 times). In contrast, there was a non-significant increase at 40% F.C (1.693 times) compared to the control. Expression of LOX2 was significantly increased in both water-stressed conditions (40% F.C and 70% F.C), with a pronounced expression at 70% F.C (1917.49-fold increase) and 40% F.C (60.129-fold increase). Expression of CYP710A1 was diminished at both 40% and 70% F.C levels when compared to the control, with 0.419- and 0.015-times changes at expression levels, respectively.

**Fig. 5:**
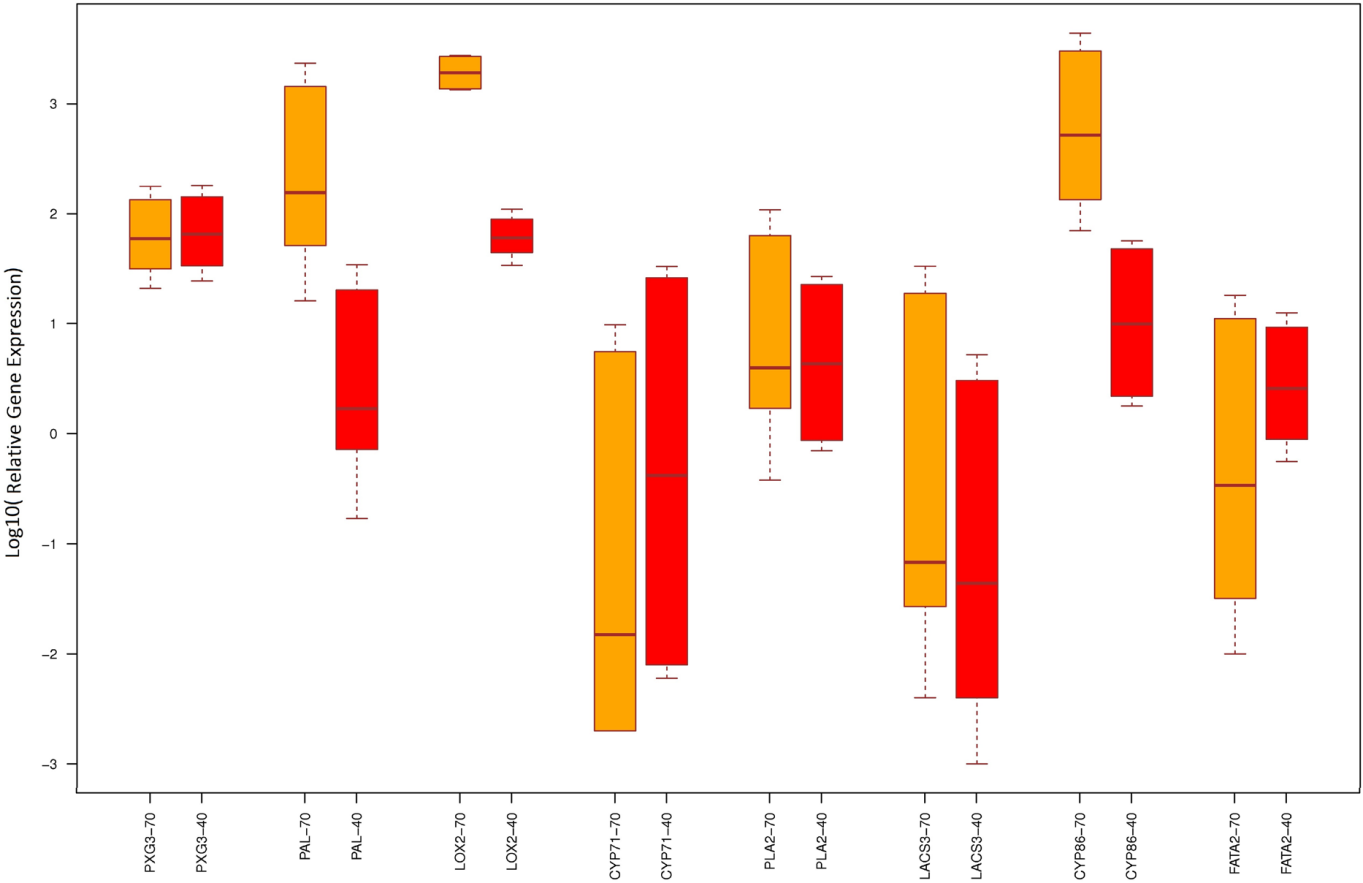
Relative expression analysis of 8 hub genes (4 up-regulated and 4 down-regulated genes) at 70%FC (orange color) and 40% FC (red color) versus FC treatment (field capacity). The relative expression of genes is shown based on Log10. Because the differences in expression between the different genes were so large, logarithms were used to make them easier to display and compare better

The expression of PLA2-ALPHA increased at 40% F.C (4.332 times) and 70% F.C (3.972 times) compared to the control, but there was no statistical significance. The LACS3 demonstrated a decreased expression at both 40% and 70% F.C (0.044 and 0.068, respectively) compared to the control condition. The CYP86A1 showed a significant up-regulation at 40% F.C and 70% F.C compared to the control, higher at 70% F.C (519.147-fold increase) than 40% F.C (9.952). The expression of the FATA2 gene showed a non-significant increase at 40% F.C (2.585 times) and a reduction at 70% F.C (0.343 times) compared to F.C (Fig. 5).

### Comparison of Silybin content ​​in different levels of drought stress

To quantify silybin a and b in plant extracts, their concentrations were calculated using regression equations for different drought stress treatments, as shown in Table 4. Our results demonstrated a statistically significant difference (*p* ≤ 0.05) in the silybin a and b levels between drought stress treatments and the control. Furthermore, silybin a and b levels increased significantly with escalating drought stress. Specifically, the severe stress treatment (40% F.C) caused a significant increase in silybin a and b levels (67.30 and 98.92 mg/g Grain DW, respectively) compared to the control and the 70% F.C treatment.

**Table 4:** Silybin a and b values calculated for different levels of water stress

Water stress treatment levels	Silybin a (mg/g Grain DW)	Silybin b (mg/g Grain DW)
Field capacity	41.23^b^	58.18^c^
70% Field capacity	41.98^b^	64.28^b^
40% Field capacity	67.30^a^	98.92^a^

### Interaction of gene expression with Silymarin levels

**Fig. 6:**
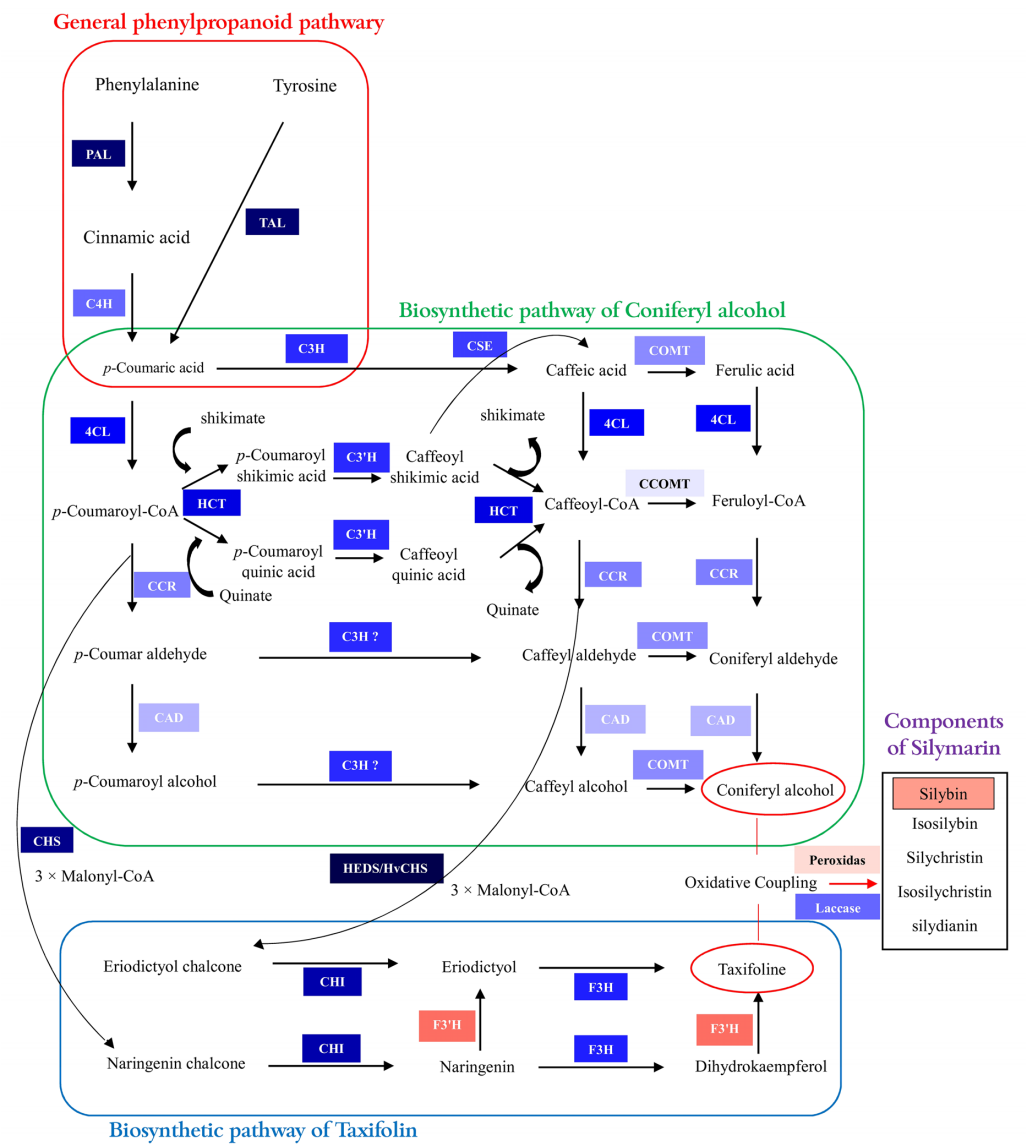
Overview of silymarin biosynthesis pathway along with gene expression. Blue colored boxes indicate a decrease in gene expression and red colored boxes indicate an increased gene expression. The intensity of the color boxes indicates the amount of increase or decrease in expression

The study investigated the expression of 19 genes directly engaged in silymarin biosynthesis. Under conditions of mild drought stress (represented as 70% F.C), an upregulation in the expression of myriad genes was observed, with the exceptions being caffeine-CoA 3-O-methyltransferase (CCOMT) and Peroxidase, both of which showed a decrease in expression levels. As drought stress intensity increased to 40% F.C, alterations in gene expression were recorded. Only two genes demonstrated an increase in expression, namely Flavonoid 3′-hydroxylase (F3′H) (164) and Peroxidase (15.14), under severe drought stress conditions, whereas a significant reduction in expression was observed for genes including Tyrosine ammonia-lyase (TAL) and Phenylalanine ammonia-lyase (PAL) (7504.8), Cinnamate 4-hydroxylase (C4H) (438.9), p-Coumarate 3-hydroxylase (C3H) and p-Coumaroylester 3′- hydroxylase (C3′H) (717.39), Caffeic acid 3-O-methyltransferase (COMT) (182), Caffeoyl-CoA 3-O-methyltransferase (CCOMT) [5], 4-Coumaric acid: Coenzyme A Ligase (4CL) (1058), Hydroxycinnamoyl-CoA Shikimate/Quinate Hydroxycinnamoyl Transferase (HCT) (1099), Caffeoyl Shikimate Esterase (CSE) (615.5), Cinnamoyl-CoA Reductase (CCR) (214), Cinnamyl Alcohol Dehydrogenase (CAD) [63], Chalcone Synthase (CHS) (5485), Homoeriodictyol/Eriodictyol Synthase (HEDS/HvCHS2) (27128), Chalcone Isomerase (CHI) (3890), Flavanone 3-Hydroxylase (F3H) (747), and Laccase (386.9) (Fig. 6). Additionally, it was observed an increase of silybin a and b content under drought stress conditions.

## Discussion

This article discussed the impact of drought stress on milk thistle plants from four different perspectives.

### What biological pathways and reactions are DEGs involved in?

Most genes were associated with the metabolic pathways and the biosynthesis of secondary metabolites, which became statistically significant at a high probability level (Table 4). The evolution of specific metabolic pathways deriving from primary metabolism is crucial for plants to interact with their surrounding environment [25, 26]. Pathways of secondary metabolites emerged from primary pathways, which indicates that the advent of novel enzymatic activities targeting primary metabolites has led to the production of new compounds. These compounds have improved plant adaptability to specific environmental conditions and have progressively evolved into specialized metabolites, as evidenced in the work of Weng [27].

After the pathways above, pathways associated with carbon metabolism and amino acid biosynthesis were identified with the highest number of genes. Plants’ ability to navigate and adapt to ever-changing environmental conditions is partly attributable to the dynamic nature of carbon metabolism. This flexibility facilitates an efficient response to fluctuations in the availability of CO2 or the products of electron transport in response to the demand for carbon assimilation [28]. Moreover, beyond its critical role in protein synthesis, amino acid metabolism is deeply interconnected with several other metabolic processes, including energy and carbohydrate metabolism, balancing the carbon-nitrogen budget, influencing hormone and secondary metabolism, and modulating responses to stress, among others.

Gene expression involved in the protein processing pathway of the endoplasmic reticulum showed a diminished expression under severe stress conditions compared to F.C and 70% F.C; conversely, genes engaged in the starch and sucrose metabolism pathways showed increased expression under severe stress, compared to F.C and 70% F.C. The requirement for protein folding within the endoplasmic reticulum can increase owing to various developmental scenarios or environmental conditions. The accumulation of proteins that are either unfolded or poorly folded within the endoplasmic reticulum triggers the unfolded protein response (UPR), a mechanism devised to mitigate the detrimental impact of such proteins in plant systems [29]. Hence, a reduced expression of genes associated with protein processing could lead to increased accumulation of misfolded or unfolded proteins within the endoplasmic reticulum, leading to stress. Starch and sucrose play crucial, albeit complementary, roles in carbon storage and transport at an organismal level, with their metabolic activities responsible for a significant portion of carbon flux across most plant species [30, 31].

Genes associated with the spliceosome pathway have played a significant role under both mild and severe stress conditions, including a high number of genes. The spliceosome, a substantial RNA-protein complex, catalyzes the excision of introns from pre-mRNAs within the nucleus. Drought conditions induce considerable alterations in the spliceosome, indicative of adaptive transcriptomic modifications and the stress response [32].

Expressions of genes involved in the oxidative phosphorylation and mRNA surveillance pathways demonstrated reductions under both severe (40% F.C) and mild (70% F.C) stress in comparison to control conditions (F.C). In Plants, ATP production is predominantly facilitated by mitochondrial oxidative phosphorylation (OXPHOS) and chloroplastic photophosphorylation (PHOTOPHOS) (Braun, 2020). Moreover, transcripts emanating from the eukaryotic nucleus undergo mRNA surveillance, a process ensuring that only flawless mRNAs proceed to translation [33].

Under severe stress, genes within the RNA degradation pathway and those involved in glycine, serine, and threonine metabolism showed increased expression compared to F.C. treatment. RNA degradation proteins are instrumental in removing superfluous RNA from the cells [34]. Threonine, a vital amino acid, features a close biochemical interconnection with methionine and isoleucine, and is convertible with glycine and serine [35, 36].

In the 40% F.C vs. 70% F.C pairwise comparison, the MAPK signaling pathway had a prominent activity. Moreover, the expression of genes engaged in ubiquitin-mediated proteolysis and fatty acid metabolism pathways was increased, whereas those associated with the RNA transport pathway declined. MAPKs are central to one of plants’ most comprehensively investigated signaling mechanisms, bridging stimulus perception with a range of cellular and adaptive responses [37]. The expression of genes of the MAPK pathway under drought stress in our experiment was consistent with the expression of genes of this pathway in the experiment of Hasanpour et al. [38]. The ubiquitin proteolytic system is critical in different cellular functions, comprising cell cycle regulation, control over signal transduction pathways, modulation of inflammatory and immune responses, and influence on development and differentiation processes [39]. Fatty acids biosynthesized in chloroplasts are immediately merged with glycerol to form a galactolipid, a primary constituent of the chloroplast membrane. They are also transported to the cytoplasm to join with glycerol within the endoplasmic reticulum, forming a cell membrane phospholipid. RNA transport denotes the active translocation of ribonucleic acid (RNA) molecules within the cell to different locations [40].

### What biological processes, molecular functions, and cellular components are DEGs involved in?

Most genes that showed upregulation or downregulation across six different groups were engaged in the biological processes of protein phosphorylation, oxidation-reduction mechanisms, and the embryo development process ending in seed dormancy (Table 5). Protein phosphorylation, as one of the most prevalent types of post-translational modifications, is integral to a myriad of cellular functions, including but not limited to the cell cycle, signal transduction pathways, and environmental stress responses [41–43]. The cyclical nature of oxidation and reduction is fundamental for the mitigation of dormancy and quiescence, facilitating the activation of the cell cycle and inducing the genetic and epigenetic controls essential for growth and differentiation in various environmental conditions [44]. During embryo development, which ends in seed dormancy, a distinct progression of the embryo is observed from the stage of zygote formation until the end of seed dormancy [45]. Since gene expression analysis was performed at the flowering stage of the plants, this suggests that the increased expression of genes associated with embryogenesis may be attributable to this developmental stage.

The molecular functions encompassing protein binding, mRNA binding, and ATP binding were distinguished by having the highest number of up- and downregulated genes. Plant RNA-binding proteins (RBPs), characterized by their heterogeneity and being ubiquitous, control gene regulation both co- and post-transcriptionally. This coordination modulates RNA metabolism in response to internal and environmental signals, thereby regulating over 60% of the plant’s transcriptome engaged in growth, development, and stress adaptation [46–51].

The ATP-binding cassette (ABC) transporters, a ubiquitous gene family across all life domains, engage in various biological processes. They have essential roles in metabolite transmembrane transport within plant cells through ATP hydrolysis and are instrumental in seed germination, lateral root development, stomatal operation, and environmental stress reactions [52–56]. The substrates for ABC proteins span across hormones, toxic substances, pigments, defense-important secondary metabolites, lipid molecules, and reactive oxygen species (ROS)-associated compounds [57–62]. The genes that were up- or downregulated showed predominant activity in the nucleus, cytoplasm, and chloroplasts, particularly under mild and severe drought stress conditions, highlighting these cellular locations as critical centers of gene activity.

### What transcription factors are DEGs related to?

Our results showed the significant involvement of transcription factors, including ERF, C3H, bHLH, BZIP, HD-ZIP, NAC, LBD, MYB-RELATED, NIN-LIKE, and WRKY, in mediating drought stress tolerance mechanisms. Within the AP2/ERF superfamily, the Ethylene Responsive Factor (ERF) subgroup has the highest number of proteins and has garnered considerable attention for its biological functionalities. These members are crucial in regulating plant resilience against a spectrum of abiotic stressors, including drought, salinity, and cold stress. Activation of ERFs typically occurs through phosphorylation by mitogen-activated protein kinases or escape from ubiquitin-ligase enzymes, subsequently facilitating their association with nucleic acid proteins before their binding to cis-regulatory elements located in the promoter regions of genes responsive to stress [63, 64].

Numerous studies have confirmed the critical roles of AP2/ERF transcription factors, particularly the ERF subfamily, in plant responses to abiotic stresses, growth regulation, and secondary metabolism. Ma et al. [65] provided a comprehensive review highlighting the involvement of AP2/ERF members in regulating stress-responsive gene expression under drought, salinity, and cold conditions. Similarly, Licausi et al. [66] and Müller and Munné-Bosch [67] emphasized the function of ERFs as key regulatory hubs at the intersection of hormonal signaling pathways—such as ethylene and abscisic acid—and environmental adaptation. In addition to their stress-related roles, Phukan et al. [68] and Baharudin and Osman [69] reported that ERF transcription factors contribute to the regulation of secondary metabolite biosynthesis, including pathways for terpenoids and flavonoids. Furthermore, Wu et al. [63] demonstrated that ERFs activate stress-responsive pathways through binding to cis-regulatory elements in the promoters of target genes. In a functional study on *Populus*, Kong et al. [70] showed that overexpression of the PtoERF15 gene enhances drought tolerance via activation of the jasmonic acid (JA) signaling pathway, leading to increased expression of defense-related genes. These studies underscore the multifaceted regulatory roles of ERF transcription factors in orchestrating plant physiological and molecular responses to adverse environmental conditions.

Regarding zinc finger proteins (ZFPs), the Cysteine3Histidine (C3H) is distinguished by a Znf motif comprising three cysteines and a single histidine residue bound to a zinc cation. This configuration is a characteristic of mRNA-binding proteins conserved across numerous eukaryotes [71, 72]. The C3H gene family has been explored in various dicots and monocots, revealing its signature motif and pivotal role in regulating plant growth, developmental processes, and response mechanisms to environments [73].

A study by Zheng et al. [74] systematically identified members of this family and examined their expression profiles under three different stress conditions: drought, heavy metal (lead) stress, and Fusarium oxysporum infection. Their results revealed that several CmC3H genes were significantly regulated in response to these stresses, suggesting a potential role in the plant’s defense mechanisms and stress tolerance. These findings provide a theoretical foundation for future research aimed at improving biotic and abiotic stress resistance in melon [74].

Among various transcription factors involved in drought tolerance, OsZFP37, OsC3H, OsNAC94, and OsbHLH148 have been shown to exhibit significantly higher transcript levels in the drought-tolerant rice cultivar IR36 compared to MTU1010 [75]. Moreover, overexpression of these transcription factors enhances drought tolerance, while their silencing increases drought sensitivity. This evidence highlights the critical roles of C3H, NAC, and bHLH family members in orchestrating drought stress responses, suggesting similar mechanisms may be conserved across different plant species [75].

Other researchers have also confirmed the positive role of transcription factors from the C3H family and related families in drought stress responses and the regulation of secondary metabolism. For instance, Arisha et al. [76] identified genes associated with drought stress responses in hexaploid sweet potato using RNA-Seq analysis. Additionally, Jiang et al. [77] investigated the C3H gene family in *Aegilops tauschii* and demonstrated their involvement in drought stress response. Furthermore, Cheng et al. [78] analyzed the C3H gene family in wheat and explored their association with seed dormancy and germination.

Furthermore, many reports showed the involvement of other transcription factors, including bHLH [79], BZIP [80], HD-ZIP [81], NAC [82], LBD [83], MYB-RELATED [84], NIN-LIKE [85], and WRKY [86] in conferring resilience to a multitude of abiotic stresses. These findings underscore the intricate regulatory networks underpinning plant adaptive responses to adverse environmental conditions.

In recent years, numerous studies have elucidated the role of basic helix-loop-helix (bHLH) transcription factors in regulating plant responses to environmental stresses and the biosynthesis of secondary metabolites. Rabeh et al. [87] presented a comprehensive review of transcription factor-mediated regulation of plant secondary metabolism under stress, highlighting the cross-talk between bHLH, MYB, and WRKY factors in stress signaling pathways. In a study focused on woody plants, Yan et al. [88] explored the functions and regulatory mechanisms of bHLH transcription factors and demonstrated their involvement in responses to salinity and low temperature. Gaude and Jalmi [89] further discussed the transcriptional regulation of stress-induced secondary metabolite biosynthesis, specifically the induction of such compounds under drought and UV exposure. Muhammad et al. [90] examined the diverse roles of bHLH genes in fleshy fruit-bearing species, showing their contributions not only to stress responses but also to developmental processes such as fruit ripening and pigmentation. In two separate reviews, Qian et al. [91] and Guo et al. [92] summarized the functions of bHLH transcription factors in tolerance to abiotic stresses, including drought, cold, and salt. Additionally, Wang et al. [93] identified StbHLH47 in potato (*Solanum tuberosum* L.) as a negative regulator of drought tolerance, underscoring the potential for both positive and negative regulatory roles of bHLH family members in stress adaptation.

### Examination of two main products of milk thistle: Silymarin and lipids

#### Silymarin

In plants, flavonolignans biosynthesis and accumulation are modulated by various environmental factors. An oxidative linkage between the phenylpropanoid derivative, coniferyl alcohol, and the flavonoid compound, taxifolin, mediates silymarin biosynthesis [94]. These two precursor molecules originate from phenylpropane and flavonoid structural units, with coniferyl alcohol and taxifolin being produced via the monolignol and flavonoid biosynthetic pathways, respectively [95].

Silymarin comprises a complex of seven flavonolignans—including silybin a, silybin b, silychristin a, silychristin b, isosilybin a, isosilybin b, and silydianin—and one flavonoid, taxifolin [94]. The compound silibinin, a combination of silybin a and b, has been shown to underlie the hepatoprotective capabilities attributed to silymarin in milk thistle [94]. Analytical quantification through HPLC demonstrated that silybinin levels in seeds of milk thistle were significantly increased under severe drought stress compared to control conditions, suggesting stress-induced phytochemical accumulation [96]. They showed that among the various components of silymarin, silybin accumulation was significantly higher in seeds collected from plants subjected to drought stress. Furthermore, this phenotypic response was reported in some studies [97–99], indicating an adaptive silymarin biosynthetic increase as a component of the antioxidative defense mechanism against prolonged water deficit stress.

Our transcriptome analysis, utilizing RNA-Seq technology, revealed an upregulated expression of silymarin biosynthesis-related genes under mild stress conditions (70% F.C) and a downregulation under severe stress (40% F.C). This was consistent with findings from ElSayed et al. [96], where the expression of genes encoding chalcone synthase (CHS1, CHS2, CHS3), integral to the silybin biosynthetic pathway, increased under drought stress conditions. These data suggested a genetic predisposition in milk thistle towards drought stress tolerance, with seeds derived from severely stressed plants showing an increased silymarin content [99]. At the enzymatic level, the CHS and chalcone isomerase (CHI) play important roles in catalyzing the biosynthesis of naringenin through the conversion of malonyl-CoA and 4-hydroxycin-namoyl-CoA molecules, a critical initial step in the phenylpropanoid pathway leading to taxifolin biosynthesis, the flavonoid precursor of silymarin [100, 101].

It was shown that expression of CHS is induced in response to various biotic and abiotic stressors [102], a phenomenon also observed in our study, which confirms the increased expression of CHS genes in milk thistle leaves under drought conditions. Another study conducted by Drouet et al. [19] has elucidated the involvement of genes such as PAL (l-phenylalanine ammonia-lyase), CAD (cinnamyl alcohol dehydrogenase), CHS, F3H (flavanone 3-dioxygenase), F3’H (flavone 3’-hydroxylase), LAC (laccases), and POX (peroxidases) in the silymarin biosynthesis pathway. Their results showed that key genes in the phenylpropanoid and flavonoid pathways, such as F3H, F3’H, CHS, and CHI, are activated during milk thistle fruit maturation and coincide with the increase in flavonolignan accumulation. Additionally, PAL and CAD genes are involved in the production of lignan precursors, while POX and LAC genes play a role in the final coupling of these compounds. These findings indicate that these genes and enzymes closely and coordinately regulate silymarin biosynthesis in the fruit pericarp. Our experimental findings were consistent with these observations, reinforcing the hypothesis of a coordinated genetic and enzymatic regulation underpinning silymarin biosynthesis.

#### Lipids

Milk thistle is recognized as an oil-producing and medicinal plant, distinguished by its rich content of unsaturated fatty acids, making it an advantageous choice for edible oil within the food industry. The role of lipids, encompassing fatty acids, hydrocarbons, esters, steroids, etc., is crucial in plant cells, especially under stress conditions such as drought. This is due to their involvement in initiating defense mechanisms and mitigation processes in response to stress factors. To assess the critical role played by milk thistle lipids in improving drought tolerance, a field study was conducted to investigate the plant’s response under different levels of water deficit: F.C, 70% F.C, and 40% F.C. The findings showed the significant function of lipids as key components of plant cellular structure, particularly in adverse conditions.

Previously, the lipid constituents and the genes responsible for their biosynthesis in milk thistle were identified. Subsequently, eight genes (CYP86A1, CYP710A1, FATA2, LACS3, LOX2, Palmitoyl-protein thioesterase (PAL), PLA2-ALPHA, and PXG3) were shown to have differential expression, were selected for further examination. The metabolic networks related to these genes were identified, revealing a correlation between the two networks. This enabled the grouping of specific lipids with their biosynthesizing genes for each pathway.

In plants, lipoxygenases (LOXs) are key enzymes in the fatty acid metabolism pathway and play crucial roles in regulating physiological and biochemical processes. These enzymes are present in various organs including roots, stems, leaves, flowers, and fruits, and significantly influence organ growth and development, fruit ripening, oil oxidation, and signal transduction related to biotic and abiotic stresses. Additionally, LOXs regulate secondary metabolism, impacting the production of important compounds such as flower aromas and fruit flavors [103]. Similarly, LACS genes, responsible for activating long-chain fatty acids, facilitate their entry into essential metabolic pathways like lipid synthesis and degradation. They contribute to forming protective layers in plants, reducing water loss and enhancing tolerance to stresses such as drought and salinity. Defects in these genes lead to increased water loss and decreased drought tolerance, highlighting their essential role in plant growth, development, and stress resilience [104]. Furthermore, PXG3, a peroxygenase with potential calcium-binding capability, is involved in the degradation of storage lipids within oil bodies and participates in abiotic stress-related signaling pathways. It may contribute to drought tolerance through stomatal regulation under water-deficient conditions [105].

The PLA2 enzyme hydrolyzes membrane phospholipids to release free fatty acids and lysophospholipids, playing a vital role in plant responses to environmental stimuli. It also regulates the trafficking of PIN proteins involved in hormonal balance and suppresses the transcriptional activity of MYB30, thereby modulating plant defense responses [106]. PAL plays a key role in terminating de novo fatty acid synthesis, particularly in producing the saturated fatty acid palmitic acid (16:0), which is critical for plant growth and seed development. It may also participate in the synthesis of long-chain fatty acids [107]. The CYP710A1 gene is crucial for lignification and enhancing drought tolerance in plants. It promotes lignin accumulation in the cell wall and regulates the expression of key structural genes involved in lignin biosynthesis, thereby strengthening cell structure and improving plant resistance to water deficit conditions [108]. Likewise, CYP86A1 is a key enzyme responsible for ω-hydroxylation of fatty acids, playing an important role in suberin biosynthesis—a protective barrier in plants. It is predominantly expressed in roots and is essential for the production of suberin monomers, contributing to enhanced plant protection against environmental stresses [109]. Finally, despite extensive investigation, no reliable information was found regarding the role of the FATA2 gene in these processes. These selected genes were further utilized to validate the expression results obtained from RNA-Seq experiments through qRT-PCR, showing consistency in gene expression outcomes.

### Conclusion

This study investigated the impact of drought stress on transcriptome (DEGs) and related biological pathways in milk thistle. Thousands of genes were identified, notably involved in flavonoid and lipid biosynthesis and stress response pathways (including MAPK). Additionally, transcription factors regulating drought-responsive genes were characterized. Metabolomic analysis revealed a significant increase in key metabolites such as silybin a and b, which are strongly correlated with gene expression changes, highlighting complex gene–metabolite interactions under drought conditions. Morphological and physiological data confirmed milk thistle’s notable drought tolerance. Optimal irrigation at 70% field capacity with four-day intervals provided the best growth and performance. This study represents one of the first de novo transcriptome analyses of milk thistle under drought stress, simultaneously examining gene expression related to lipid and flavonolignan pathways. The results offer practical agricultural strategies and provide deeper insights into the mechanisms of drought tolerance.

## Materials and methods

**Fig. 7:**
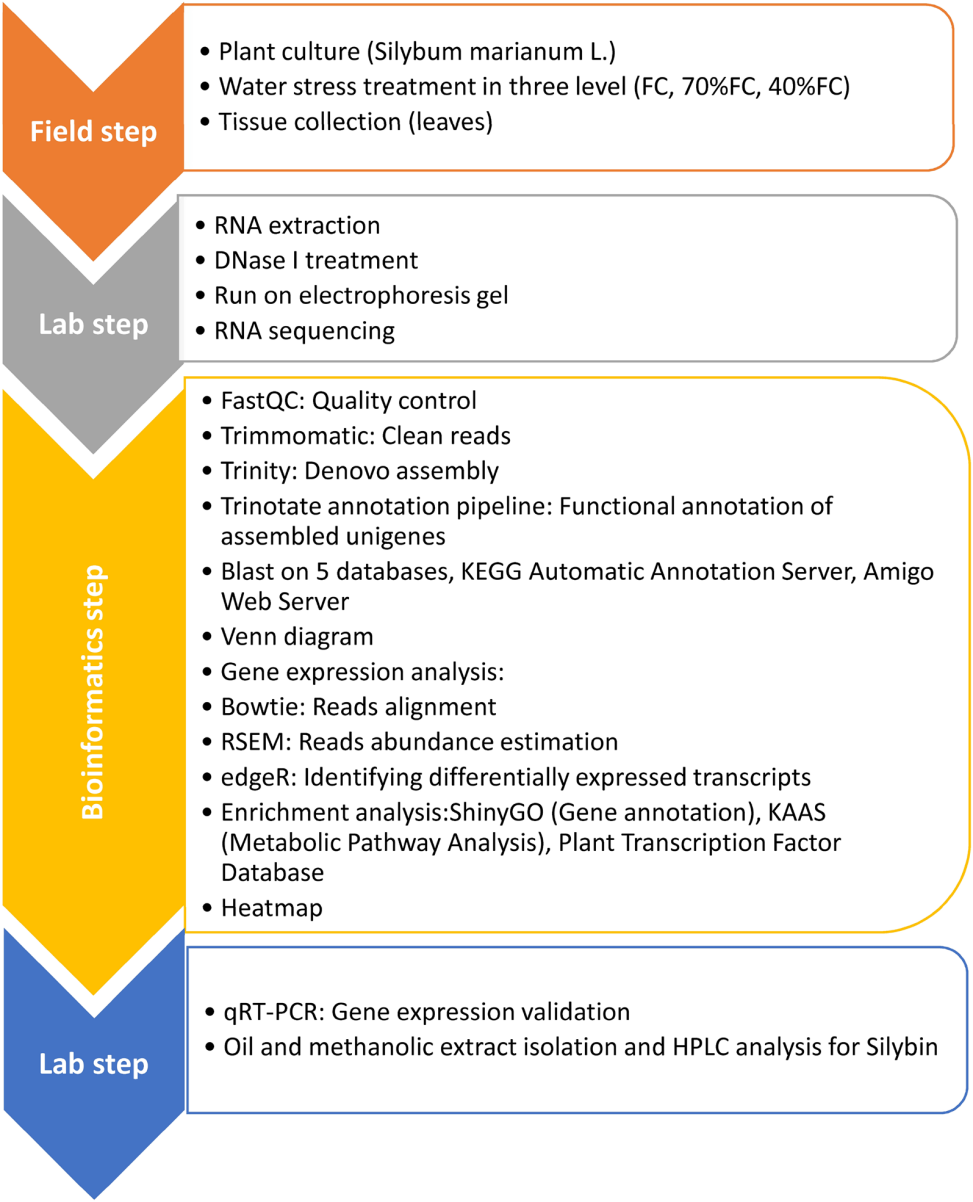
Field and Lab experiment and RNA-seq analysis workflow

The general process of doing this article is presented in Fig. 7. All bioinformatics analyses were conducted using tools and databases that were available and accessed between 2019 and 2020.

### Plant materials and experimental site

The milk thistle seeds were bought from Pakanbazr, Isfahan, Iran. The research site was at Shahid Beheshti University in Tehran, Iran (latitude 51.23°N, longitude 35.48°E). The area had a mountainous and moderate climate, with an average annual temperature and precipitation of 22 °C and 145.2 mm, respectively. Monthly data on precipitation, air temperature, moisture, and wind speed were gathered from the meteorological site (Table 5). The soil consisted of one-third clay, one-third sand, and one-third leaf compost. The total area of the field encompassed 150.0 m^2^. Furrows were carefully established for the study, and the spacing of plants within the rows was set at 0.5 m, with a 1-meter gap between the rows [110, 111].

**Table 5:** Atmospheric information of Tehran (Shemiranat) from 2017.03.21 to 2017.07.22

Atmospheric information	From 2017.03.21 To 2017.04.20	From 2017.04.21 To 2017.05.21	From 2017.05.22 To 2017.06.21	From 2017.06.22 To 2017.07.22
Rainfall (mm)	60.5	76.7	0.0	26.3
Mean temperature (°C)	12.7	19.6	25.8	28.1
Moisture (%)	53	41	19	28
Wind speed (mps)	10.0	17.0	12.0	10.0

### Field experiments and drought stress treatments

The experiment began with sowing milk thistle seeds, followed by regular irrigation every two days. When the plants reached the flowering stage, they were exposed to drought stress using a weighted method. During this period, the plants received irrigation at 100% field capacity (F.C) bi-daily, 70% F.C once every 4 days, and 40% F.C every 8 days [112]. After 8 days, leaf sampling was conducted. Four plants (four biological replicates) were randomly selected, and medium-sized leaves from the middle part of the stem were collected. These leaves were quickly wrapped in aluminum foil, immersed in liquid nitrogen, and kept in a laboratory freezer at −80 °C.

### RNA-Seq library construction and sequencing

The RNA was isolated from milk thistle leaves (three biological replicates) using the RNeasy Plant Mini Kit (QIAGEN, USA), following the protocols provided by the manufacturer. The isolated RNAs were subjected to DNase I treatment (Thermo Fisher Scientific, USA) to eliminate any potential DNA contamination. The 1% agarose gel and a NanoDrop 1000 spectrophotometer (Thermo Scientific, USA) were employed to evaluate the quality and concentration of the RNA samples, respectively. Further assessment of RNA quality was achieved using a QC Bioanalyzer (Agilent Technologies, BGI-Hong Kong NGS Lab), ensuring that the RNA integrity number (RIN) for each sample exceeded 7. The procedure for selecting Poly A, preparing cDNA, ligating adapters, forming clusters, and sequencing was executed at the Beijing Genomes Institute (China), following the manufacturer’s protocols, utilizing standard Illumina sequencing kits. The sequencing process employed the Poly A mRNA Capture technique on the BGISEQ-500 platform, using a paired-end strategy with a read length of 100 bp and generating 6Gbp of raw data.

### Processing of RNA-Seq data and de Novo assembly

The FastQC tool [113] available at http://www.bioinformatics.babraham.ac.uk/projects/fastqc/ assessed the quality of the initially sequenced reads. Subsequent trimming of low-quality segments and adaptor sequences from these reads was accomplished using the Trimmomatic software (Version 0.39) [114]. The quality control process post-trimming was once again verified through the application of FastQC, ensuring the retention of only high-quality reads. These processed reads were then pooled for de novo assembly, which was executed using the Trinity software (Release 2021-03-04) [115], according to the default parameters while setting the k-mer length at 31. The CAP3 package was employed to cluster the transcripts based on a 95% identity threshold and a minimum contiguous sequence length of 200 nucleotides to refine the assembly output further. The identification of potential coding sequence (CDS) regions within the sequenced transcripts was facilitated using the TransDecoder tool (http://transdecoder.github.io), which was pivotal for predicting open reading frames (ORFs). The concluding stage involved leveraging the protein sequences derived from these predicted ORFs to delineate unigenes, enriching the understanding of the transcriptome’s coding potential.

### Functional annotation of milk Thistle assembled unigenes

The Trinotate annotation pipeline (http://trinotate.github.io/) was utilized to annotate the milk thistle transcriptome. This process included subjecting the assembled milk thistle unigenes to BLAST analysis against several databases: non-redundant nucleotide (NT) (http://www.ncbi.nlm.nih.gov/), non-redundant proteins (NR) (http://www.ncbi.nlm.nih.gov/), UniProt (Swiss-Prot and TrEMBL) [116], Arabidopsis (version TAIR11) [117], Sunflower protein (http://ftp.ensemblgenomes.org/pub/plants/release-50/fasta/helianthus_annuus/pep/) database with an E-value threshold of 10e-5 for significance. To identify metabolic pathways associated with each unigene, Kyoto Encyclopedia of Genes and Genome (KEGG, http://www.genome.jp/kegg/) analysis was performed [118] using KEGG Automatic Annotation Server (KAAS, http://www.genome.jp/kegg/kaas/) [119]. For the functional classification of assembled unigenes, the Amigo Web Server (http://amigo.geneontology.org) was employed, following the established protocols [120]. Venn diagrams were generated through the InteractiVenn website based on gene trinity ID [121], facilitating a visual representation of our findings.

### Differential gene expression analysis

The quantification of transcripts assembled by Trinity was performed by employing the RSEM (version 1.3.3) [122]. Initially, high-quality sequence reads were realigned to the constructed transcriptome via Bowtie2 (version 2.4.1) [115]. Subsequently, RSEM estimated the read alignment of individual transcripts. The output files from RSEM encompassed normalized expression metrics for each unique transcript and gene. The identification of differentially expressed (DE) contigs was achieved through analysis of the counts matrix generated by RSEM, utilizing the edgeR package from Bioconductor [123] in Rstudio [124]. Within this analysis, TMM normalization was applied by the edgeR package to account for variances in sample composition. The criteria for obtaining DEGs included a threshold false discovery rate (FDR) of ≤ 0.001, alongside a requisite absolute Log ratio ≥ 2 and a minimum four-fold expression change.

### Enrichment analysis

The analysis of DEGs involved singular enrichment analysis (SEA) through the ShinyGO platform (http://bioinformatics.sdstate.edu/go/) for the identification of enriched gene ontologies (GO). These DEGs were systematically categorized into three main groups: bioloical processes (BP), molecular functions (MF), and cellular components (CC), with a significant threshold determined by the p-value. Further, the Kyoto Encyclopedia of Genes and Genomes (KEGG) pathways were determined by employing in-house scripts developed by the NIGEB Genome Center, which leveraged both the David and KEGG databases for comprehensive analysis. To identify transcription factors, the analysis turned to the resources of the Plant Transcription Factor Database (http://planttfdb.gao-lab.org/).

### Validation of RNA-seq data using qRT-PCR

#### RNA extraction and cDNA synthesis

Total RNA was isolated from 200 mg of mature milk thistle seeds collected at the flowering phase, utilizing a total RNA extraction kit (RB1001, RNA, Iran). Then, DNase I (RB125A, RNA, Iran) was used to eliminate any potential genomic DNA contamination from isolated RNA. The quality and concentration of the isolated RNA were evaluated using 1% agarose gel electrophoresis and spectroscopic analysis with a NanoDrop 1000 spectrophotometer (Thermo Scientific, USA), respectively. Subsequently, five µg of the purified RNA was employed to synthesize cDNA using a cDNA Synthesis kit (RB125A, RNA, Iran), according to the guidelines provided by the manufacturer. Then, the synthesized cDNA was stored at −20 °C for future experimentation.

#### Primer design and qRT-PCR analysis

Primers for targeted genes were designed by employing the nucleotide sequences of closely related species, *Carthamus tinctorius* and *Helianthus annuus*, from the NCBI database. Subsequent comparative analysis was performed against our prior milk thistle assembly data employing the BlastStation tool (https://www.blaststation.com/), enabling the selection of sequences with high identity. Regions proximal to the terminal polyadenine sequences, targeting a sequence length between 150 and 250 base pairs were considered for designing primers. Parameters such as homodimer and heterodimer formation, stem-loop structures, GC content, and melting temperature (TM) were meticulously evaluated utilizing both Oligo 7 software (https://www.oligo.net/) [125] and Vector NTI^®^ Express Designer Software (https://vector-nti.software.informer.com/11.0/) [126]. The 18 S rRNA gene was used as the reference gene for normalization, and its suitability had been previously tested and validated in our prior experiments. Ultimately, the synthesis of designed primers was carried out by Bioneer Company (South Korea). Detailed sequences and additional characteristics of the primers are documented in Table 6.

**Table 6:** RT-qPCR primer sequence of selected candidate and reference genes and their amplification characteristics

Primer name	Primer sequence	PCR product length (bp)	TM	PCR amplification efficiency
*PXG3*	F: CCAGCAAACCTTGAGAAC	207	54	1.95
	R: GCAACGCCTTACTGATTC		54	
*PAL*	F: AGGGTAATCTAATAGGCC	155	52	1.88
	R: ACTGAACTCTCCATCTGG		54	
*LOX2*	F: CCACAGTGGAAACATGTC	255	54	1.94
	R: ATCTTCAACCGCCATACC		54	
*CYP710A1*	F: TTCTACCTACACTGAGC	198	50	1.90
	R: AGGAAGTCAAACAGGTGG		54	
*PLA2-ALPHA*	F: ATGGGAAGTACTGTGGG	194	52	1.99
	R: GTGTTGCCTTTGAATGTC		52	
*LACS3*	F: GAGATGAATTATGACGCC	256	52	1.91
	R: GCCACATATTCTCCTTG		50	
*CYP86A1*	F: ACGTGACACCTCCTCCG	242	57	1.97
	R: CATGTTCGTGGTCCAGCG		58	
*FATA2*	F: GTACTAGACGTGATTGG	177	50	2.00
	R: CTTCTGGAAATGCTAATC		49	
*18SrRNA*	F: ATGATAACTCGACGGATCGC	200	56	1.97
	R: CTTGGATGTGGTAGCCGTTT		57	

The amplification was conducted utilizing the Rotor-Gene 2000 apparatus (Corbett Life Science, Australia) employing SYBR^®^ Green Real-Time PCR Master Mix (RB120, RNA, Iran). The reaction volume (20 µl) consisted of 2× SYBR Green mix (10 µL), synthesized cDNA (1 µL), forward and reverse primers (each one µL) at a concentration of 20 nmol, and up to 7 µL of RNase-free water. The thermal cycling conditions initiated with a pre-denaturation step at 95 °C for 5 min, followed by 35 cycles of amplification, which included denaturation at 95 °C for 1 min, annealing between 50 and 60 °C based on the melting temperatures of the primers for 1 min, and extension at 72 °C for 15 s. A melting curve analysis post-amplification was executed to verify the specificity of the PCR amplifications, spanning a temperature interval between 60 and 95 °C for every reaction. Cycle threshold (Ct) values and PCR efficiency were calculated using the LinRegPCR software [127]. The relative expression levels of target genes were determined using the Relative Expression Software Tool (REST), in adherence to the protocols outlined by Pfaffl et al. [128], with the efficiency of the primers (E) incorporated into the calculation. The qRT-PCR experiments were executed in triplicate, encompassing both biological and technical replicates to ensure the reliability and reproducibility of the findings.

$$\:Gene\:expression\:ration={\left({E}_{Gene}\right)}^{\varDelta\:ct\:Gene}/{\left({E}_{Ref}\right)}^{\varDelta\:ct\:Ref}$$

### Isolation of oil and methanolic extract and Silybin measurement by HPLC

The seed samples (three biological replicates), upon drying, were finely ground into powder form. Subsequently, 10 g of this powder was used for oil extraction in a Soxhlet extractor utilizing n-hexane as the extraction solvent. This procedure was conducted at a temperature of 70 °C over six hours, and the extracted oil was subsequently stored in an amber glass container to protect against light degradation. After removing the oil, the residual powder was dried at 37 °C for one week in preparation for the methanolic extraction phase. For the extraction with methanol, 2 g of the dried, oil-free powder was combined with 200 ml of 80% methanol and subjected to agitation on a shaker for two days. Then, the resultant mixture was filtered using a filter paper and kept at a temperature of 4 °C. This extraction process was done again for the remaining residue, and the subsequent extracts were added to the first collected portion and allowed to undergo evaporation at room temperature for two weeks to yield a concentrated form.

The concentrated extracts and standard silymarin at specific concentrations were solubilized in methanol. These solutions were then introduced into the HPLC system (comprising an Agilent Infinity1260, equipped with UV/VIS and Diode Array detectors, a Merck Hitachi L-7100 pump, and an EZ Chrome software) using syringe filters of 0.2 μm pore size. A volume of 20 µl of each Silibin solution and plant sample extract was analyzed within the HPLC system. The outcome of this analytical procedure involved the examination of HPLC data and the identification of related peaks.

### Identification of Silymarin biosynthesis pathway genes

To identify the genes engaged in the silymarin biosynthesis, this study began with a comprehensive review of pathways previously described in existing literature [19, 95, 129–131]. After identifying candidate genes, their sequences were extracted from plants within the same family as Milk Thistle using NCBI and Uniprot databases. Subsequently, these sequences were blasted in the Milk thistle database (created and developed in this project) using the BlastStation tool. To validate the accuracy of the sequences, a further comparative analysis was conducted on the NCBI platform. The next step involved determining the expression levels of these identified genes through analysis of our RNA-SEQ data. Finally, the open reading frame (ORF) sequences of the genes of interest were determined by employing Vector NTI software in preparation for submission to the NCBI database.

### Statistical analysis

The analysis of the data was conducted utilizing R version 3.5.3 (https://www.r-project.org/) [132] and RStudio version 1.1.463 (https://www.rstudio.com/) [133]. We adopted a Completely Randomized Block Design (CRBD) approach, wherein we categorized the treatments as fixed effects and positioned the replications as a random effect within the ANOVA analysis. The Duncan test, accessed through the agricolae package, was employed at a 5% significance level to assess mean differences. A Completely Randomized Design (CRD) was used for metabolite and molecular data. Analysis of variance (ANOVA) was performed, and the mean comparisons were conducted using the Least Significant Difference (LSD) test at *p* ≤ 0.05 using the *agricolae* package in R.

## Supplementary Information

Below is the link to the electronic supplementary material.

Supplementary Material 1

Supplementary Material 2

Supplementary Material 3

## Data Availability

On August 26, 2020, the University of Mohaghegh Ardabili submitted the raw transcriptome sequences of Milk thistle to the SRA section of the NCBI (National Center for Biotechnology Information) international database. The Project ID was PRJNA659420, the sample ID was SAMN15905480, and the experiment accession was SRR12539246, SRR12539245, and SRR12539244 for F.C, 70% F.C, and 40% F.C, respectively. Additionally, the NCBI website received the sequence of 19 genes of the silymarin pathway identified in Silybum marianum L., along with their accessions. The names of the genes along with their accession numbers are as follows: C4H (PP965187), C3H (PP965188), COMT (PP965189), CCOMT (PP965190), 4CL (PP965191), HCT (PP965192), CSE (PP965193), CCR (PP965194), CAD (PP965195), CHS1 (PP965196), CHS2 (PP965197), CHS3 (PP965198), CHI (PP965199), F3H (PP965200), lac (PP965201), HEDS/HvCHS2 (PP965202), F3’H (PP965203) and POX (PP965204). Additionally, the sequences of lipid biosynthesis pathway genes include CYP86A1, CYP710A1, FATA2, LACS3, LOX2, Palmitoyl-protein thioesterase (PAL), PLA2-ALPHA, and PXG3 were submitted to the NCBI international database (nucleotide section) under the accession numbers of MW151571, MW151572, MW151573, MW151574, MW151575, MW151576, MW151577, and MW151578, respectively.

## Code availability

In the manuscript, we detailed a compilation of utilized software for de novo transcriptome assembly, as well as for pre- and post-assembly processes and transcriptome annotation. Each software program, along with its corresponding version, is meticulously cataloged within the Methods section. It is noteworthy that our research exclusively incorporated software that is publicly accessible, avoiding the use of any custom-developed code.
