# Supplementary material for: De novo transcriptome analysis and functional annotation of Silybum Marianum L. under drought stress with a focus on Silymarin synthesis and MAPK signaling pathways
Source: BMC Plant Biol. 2025 Aug 28;25:1150. doi: 10.1186/s12870-025-07272-5 (PMC12392515; doi:10.1186/s12870-025-07272-5)
Supplement: Supplementary file 1 — Supplementary Material 1 [file 12870_2025_7272_MOESM1_ESM.docx]

Sup1. Gene ontology functional classification of Milk thistle genes.

|  | **Gene Ontology** | **Genes No.** | **P-Value** |
| --- | --- | --- | --- |
| **Biological process** | cellular process | 12113 | 4.76E-113 |
|  | metabolic process | 8552 | 4.51E-60 |
|  | organic substance metabolic process | 7939 | 1.33E-54 |
|  | primary metabolic process | 7302 | 4.17E-50 |
|  | nitrogen compound metabolic process | 6217 | 2.26E-42 |
|  | biological regulation | 5976 | 2.98E-22 |
|  | response to stimulus | 5542 | 1.02E-37 |
|  | macromolecule metabolic process | 5532 | 5.41E-36 |
|  | protein metabolic process | 3540 | 1.17E-22 |
|  | response to stress | 3201 | 1.01E-28 |
| **Molecular function** | binding | 10003 | 7.89E-66 |
|  | catalytic activity | 8241 | 7.68E-42 |
|  | protein binding | 5503 | 8.06E-56 |
|  | organic cyclic compound binding | 5009 | 1.00E-09 |
|  | heterocyclic compound binding | 4971 | 1.04E-09 |
|  | nucleic acid binding | 3521 | 3.87E-07 |
|  | transferase activity | 3480 | 1.20E-15 |
|  | ion binding | 2720 | 1.98E-09 |
|  | hydrolase activity | 2637 | 5.60E-09 |
|  | catalytic activity, acting on a protein | 2540 | 8.37E-26 |
| **Cellular component** | cellular anatomical entity | 24378 | 1.38E-26 |
|  | intracellular anatomical structure | 21409 | 1.11E-22 |
|  | organelle | 19730 | 2.37E-18 |
|  | intracellular organelle | 19681 | 9.24E-19 |
|  | membrane-bounded organelle | 19523 | 1.07E-16 |
|  | intracellular membrane-bounded organelle | 19466 | 6.90E-17 |
|  | cytoplasm | 14684 | 1.78E-32 |
|  | plastid | 5409 | 7.86E-10 |
|  | chloroplast | 5004 | 2.62E-06 |
|  | membrane | 4923 | 4.53E-50 |
| **Protein class** | metabolite interconversion enzyme | 4000 | 5.76E-04 |
|  | protein modifying enzyme | 1740 | 1.67E-05 |
|  | transporter | 1109 | 4.04E-15 |
|  | membrane traffic protein | 367 | 2.19E-06 |
|  | transmembrane signal receptor | 323 | 3.25E-15 |
|  | primary active transporter | 290 | 2.28E-04 |
|  | secondary carrier transporter | 271 | 1.68E-02 |
|  | deacetylase | 28 | 4.67E-02 |
