# Supplementary material for: De novo transcriptome analysis and functional annotation of Silybum Marianum L. under drought stress with a focus on Silymarin synthesis and MAPK signaling pathways
Source: BMC Plant Biol. 2025 Aug 28;25:1150. doi: 10.1186/s12870-025-07272-5 (PMC12392515; doi:10.1186/s12870-025-07272-5)
Supplement: Supplementary file 2 — Supplementary Material 2 [file 12870_2025_7272_MOESM2_ESM.docx]

Sup2. KEGG functional pathway of Milk thistle genes

| **KO ID** | **Pathway** | **Gene number** |
| --- | --- | --- |
| 00010 | Glycolysis / Gluconeogenesis | 29 |
| 00020 | Citrate cycle (TCA cycle) | 18 |
| 00030 | Pentose phosphate pathway | 15 |
| 00040 | Pentose and glucuronate interconversions | 12 |
| 00051 | Fructose and mannose metabolism | 15 |
| 00052 | Galactose metabolism | 11 |
| 00053 | Ascorbate and aldarate metabolism | 14 |
| 00500 | Starch and sucrose metabolism | 27 |
| 00520 | Amino sugar and nucleotide sugar metabolism | 28 |
| 00620 | Pyruvate metabolism | 25 |
| 00630 | Glyoxylate and dicarboxylate metabolism | 27 |
| 00640 | Propanoate metabolism | 14 |
| 00650 | Butanoate metabolism | 9 |
| 00660 | C5-Branched dibasic acid metabolism | 2 |
| 00562 | Inositol phosphate metabolism | 22 |
| 00190 | Oxidative phosphorylation | 54 |
| 00195 | Photosynthesis | 31 |
| 00196 | Photosynthesis - antenna proteins | 9 |
| 00710 | Carbon fixation in photosynthetic organisms | 21 |
| 00720 | Carbon fixation pathways in prokaryotes | 10 |
| 00680 | Methane metabolism | 14 |
| 00910 | Nitrogen metabolism | 9 |
| 00920 | Sulfur metabolism | 11 |
| 00061 | Fatty acid biosynthesis | 12 |
| 00062 | Fatty acid elongation | 7 |
| 00071 | Fatty acid degradation | 11 |
| 00073 | Cutin, suberine and wax biosynthesis | 4 |
| 00100 | Steroid biosynthesis | 11 |
| 00140 | Steroid hormone biosynthesis | 1 |
| 00561 | Glycerolipid metabolism | 19 |
| 00564 | Glycerophospholipid metabolism | 23 |
| 00565 | Ether lipid metabolism | 5 |
| 00600 | Sphingolipid metabolism | 9 |
| 00590 | Arachidonic acid metabolism | 6 |
| 00591 | Linoleic acid metabolism | 4 |
| 00592 | Linolenic acid metabolism | 15 |
| 01040 | **Biosynthesis of unsaturated fatty acids** | 8 |
| 00230 | Purine metabolism | 26 |
| 00240 | Pyrimidine metabolism | 15 |
| 00250 | Alanine, aspartate and glutamate metabolism | 16 |
| 00260 | Glycine, serine and threonine metabolism | 22 |
| 00270 | Cysteine and methionine metabolism | 31 |
| 00280 | Valine, leucine and isoleucine degradation | 14 |
| 00290 | Valine, leucine and isoleucine biosynthesis | 6 |
| 00300 | Lysine biosynthesis | 3 |
| 00310 | Lysine degradation | 10 |
| 00220 | Arginine biosynthesis | 11 |
| 00330 | Arginine and proline metabolism | 16 |
| 00340 | Histidine metabolism | 8 |
| 00350 | Tyrosine metabolism | 12 |
| 00360 | Phenylalanine metabolism | 9 |
| 00380 | Tryptophan metabolism | 12 |
| 00400 | Phenylalanine, tyrosine and tryptophan biosynthesis | 13 |
| 00410 | Alanine metabolism | 12 |
| 00430 | Taurine and hypotaurine metabolism | 3 |
| 00440 | Phosphonate and phosphinate metabolism | 1 |
| 00450 | Selenocompound metabolism | 7 |
| 00460 | Cyanoamino acid metabolism | 10 |
| 00470 | D-Amino acid metabolism | 1 |
| 00480 | Glutathione metabolism | 14 |
| 00510 | N-Glycan biosynthesis | 14 |
| 00513 | Various types of N-glycan biosynthesis | 11 |
| 00514 | Other types of O-glycan biosynthesis | 3 |
| 00531 | Glycosaminoglycan degradation | 4 |
| 00563 | Glycosylphosphatidylinositol (GPI)-anchor biosynthesis | 12 |
| 00603 | Glycosphingolipid biosynthesis - globo and isoglobo series | 2 |
| 00604 | Glycosphingolipid biosynthesis - ganglio series | 1 |
| 00540 | Lipopolysaccharide biosynthesis | 3 |
| 00541 | O-Antigen nucleotide sugar biosynthesis | 6 |
| 00550 | Peptidoglycan biosynthesis | 1 |
| 00511 | Other glycan degradation | 6 |
| 00730 | Thiamine metabolism | 8 |
| 00740 | Riboflavin metabolism | 8 |
| 00750 | Vitamin B6 metabolism | 4 |
| 00760 | Nicotinate and nicotinamide metabolism | 10 |
| 00770 | Pantothenate and CoA biosynthesis | 11 |
| 00780 | Biotin metabolism | 6 |
| 00790 | Folate biosynthesis | 13 |
| 00670 | One carbon pool by folate | 8 |
| 00830 | Retinol metabolism | 2 |
| 00860 | Porphyrin metabolism | 19 |
| 00130 | Ubiquinone and other terpenoid-quinone biosynthesis | 18 |
| 00900 | Terpenoid backbone biosynthesis | 17 |
| 00902 | Monoterpenoid biosynthesis | 2 |
| 00909 | Sesquiterpenoid and triterpenoid biosynthesis | 6 |
| 00904 | Diterpenoid biosynthesis | 4 |
| 00906 | Carotenoid biosynthesis | 12 |
| 00905 | Brassinosteroid biosynthesis | 3 |
| 00981 | Insect hormone biosynthesis | 1 |
| 00908 | Zeatin biosynthesis | 5 |
| 00903 | Limonene and pinene degradation | 2 |
| 00281 | Geraniol degradation | 2 |
| 01051 | Biosynthesis of ansamycins | 1 |
| 01053 | Biosynthesis of siderophore group nonribosomal peptides | 1 |
| 00940 | Phenylpropanoid biosynthesis | 14 |
| 00945 | Stilbenoid, diarylheptanoid and gingerol biosynthesis | 4 |
| 00941 | **Flavonoid biosynthesis** | 8 |
| 00944 | Flavone and flavonol biosynthesis | 1 |
| 00901 | Indole alkaloid biosynthesis | 1 |
| 00950 | Isoquinoline alkaloid biosynthesis | 6 |
| 00960 | Tropane, piperidine and pyridine alkaloid biosynthesis | 6 |
| 00965 | Betalain biosynthesis | 2 |
| 00966 | Glucosinolate biosynthesis | 2 |
| 00261 | Monobactam biosynthesis | 2 |
| 00521 | Streptomycin biosynthesis | 3 |
| 00524 | Neomycin, kanamycin and gentamicin biosynthesis | 1 |
| 00401 | Novobiocin biosynthesis | 1 |
| 00405 | Phenazine biosynthesis | 1 |
| 00333 | Prodigiosin biosynthesis | 2 |
| 00254 | Aflatoxin biosynthesis | 1 |
| 00999 | Biosynthesis of various plant secondary metabolites | 9 |
| 00362 | Benzoate degradation | 2 |
| 00627 | Aminobenzoate degradation | 2 |
| 00364 | Fluorobenzoate degradation | 1 |
| 00625 | Chloroalkane and chloroalkene degradation | 2 |
| 00361 | Chlorocyclohexane and chlorobenzene degradation | 1 |
| 00623 | Toluene degradation | 1 |
| 00643 | Styrene degradation | 3 |
| 00791 | Atrazine degradation | 1 |
| 00930 | Caprolactam degradation | 1 |
| 00626 | Naphthalene degradation | 1 |
| 00980 | Metabolism of xenobiotics by cytochrome P450 | 3 |
| 00982 | Drug metabolism - cytochrome P450 | 3 |
| 00983 | Drug metabolism - other enzymes | 8 |
| 03020 | RNA polymerase | 19 |
| 03022 | Basal transcription factors | 16 |
| 03040 | Spliceosome | 51 |
| 03010 | Ribosome | 93 |
| 00970 | Aminoacyl-tRNA biosynthesis | 18 |
| 03013 | Nucleocytoplasmic transport | 40 |
| 03015 | RNA surveillance pathway | 40 |
| 03008 | Ribosome biogenesis in eukaryotes | 26 |
| 03060 | Protein export | 12 |
| 04141 | Protein processing in endoplasmic reticulum | 56 |
| 04130 | SNARE interactions in vesicular transport | 12 |
| 04120 | Ubiquitin mediated proteolysis | 40 |
| 04122 | Sulfur relay system | 5 |
| 03050 | Proteasome | 22 |
| 03018 | RNA degradation | 27 |
| 03030 | DNA replication | 12 |
| 03410 | Base excision repair | 14 |
| 03420 | Nucleotide excision repair | 18 |
| 03430 | Mismatch repair | 11 |
| 03440 | Homologous recombination | 17 |
| 03450 | Non-homologous end-joining | 4 |
| 03460 | Fanconi anemia pathway | 15 |
| 03250 | Viral life cycle - HIV-1 | 8 |
| 02010 | ABC transporters | 6 |
| 03070 | Bacterial secretion system | 5 |
| 02020 | Two-component system | 10 |
| 04010 | MAPK signaling pathway | 6 |
| 04013 | MAPK signaling pathway - fly | 9 |
| 04016 | MAPK signaling pathway - plant | 27 |
| 04011 | MAPK signaling pathway - yeast | 7 |
| 04012 | ErbB signaling pathway | 2 |
| 04014 | Ras signaling pathway | 7 |
| 04015 | Rap1 signaling pathway | 3 |
| 04310 | Wnt signaling pathway | 13 |
| 04330 | Notch signaling pathway | 2 |
| 04340 | Hedgehog signaling pathway | 4 |
| 04341 | Hedgehog signaling pathway - fly | 6 |
| 04350 | TGF-beta signaling pathway | 7 |
| 04390 | Hippo signaling pathway | 6 |
| 04391 | Hippo signaling pathway - fly | 6 |
| 04392 | Hippo signaling pathway - multiple species | 1 |
| 04370 | VEGF signaling pathway | 3 |
| 04371 | Apelin signaling pathway | 13 |
| 04630 | JAK-STAT signaling pathway | 3 |
| 04064 | NF-kappa B signaling pathway | 3 |
| 04668 | TNF signaling pathway | 1 |
| 04066 | HIF-1 signaling pathway | 15 |
| 04068 | FoxO signaling pathway | 14 |
| 04020 | Calcium signaling pathway | 7 |
| 04070 | Phosphatidylinositol signaling system | 17 |
| 04072 | Phospholipase D signaling pathway | 7 |
| 04071 | Sphingolipid signaling pathway | 12 |
| 04024 | AMP signaling pathway | 6 |
| 04022 | GMP-PKG signaling pathway | 5 |
| 04151 | PI3K-Akt signaling pathway | 17 |
| 04152 | AMPK signaling pathway | 21 |
| 04150 | TOR signaling pathway | 19 |
| 04075 | Plant hormone signal transduction | 35 |
| 04080 | Neuroactive ligand-receptor interaction | 1 |
| 04144 | Endocytosis | 37 |
| 04145 | Phagosome | 22 |
| 04142 | Lysosome | 24 |
| 04146 | Peroxisome | 23 |
| 04140 | Autophagy - animal | 22 |
| 04138 | Autophagy - yeast | 33 |
| 04136 | Autophagy - other | 13 |
| 04137 | Mitophagy - animal | 14 |
| 04139 | Mitophagy - yeast | 12 |
| 04110 | Cell cycle | 18 |
| 04111 | Cell cycle - yeast | 15 |
| 04112 | Cell cycle - Caulobacter | 4 |
| 04113 | Meiosis - yeast | 14 |
| 04114 | Oocyte meiosis | 17 |
| 04210 | Apoptosis | 8 |
| 04214 | Apoptosis - fly | 9 |
| 04215 | Apoptosis - multiple species | 3 |
| 04216 | Ferroptosis | 5 |
| 04217 | Necroptosis | 12 |
| 04115 | p53 signaling pathway | 8 |
| 04218 | Cellular senescence | 16 |
| 04510 | Focal adhesion | 5 |
| 04520 | Adherens junction | 5 |
| 04530 | Tight junction | 16 |
| 04540 | Gap junction | 2 |
| 04550 | Signaling pathways regulating pluripotency of stem cells | 1 |
| 02024 | Quorum sensing | 11 |
| 05111 | Biofilm formation - Vibrio cholerae | 1 |
| 02025 | Biofilm formation - Pseudomonas aeruginosa | 1 |
| 02026 | Biofilm formation - Escherichia coli | 3 |
| 02040 | Flagellar assembly | 1 |
| 04810 | Regulation of actin cytoskeleton | 12 |
| 04611 | Platelet activation | 1 |
| 04613 | Neutrophil extracellular trap formation | 11 |
| 04620 | Toll-like receptor signaling pathway | 1 |
| 04624 | Toll and Imd signaling pathway | 3 |
| 04621 | NOD-like receptor signaling pathway | 10 |
| 04622 | RIG-I-like receptor signaling pathway | 3 |
| 04623 | Cytosolic DNA-sensing pathway | 8 |
| 04625 | C-type lectin receptor signaling pathway | 2 |
| 04650 | Natural killer cell mediated cytotoxicity | 2 |
| 04612 | Antigen processing and presentation | 10 |
| 04660 | T cell receptor signaling pathway | 2 |
| 04658 | Th1 and Th2 cell differentiation | 1 |
| 04659 | Th17 cell differentiation | 3 |
| 04657 | IL-17 signaling pathway | 4 |
| 04662 | B cell receptor signaling pathway | 2 |
| 04664 | Fc epsilon RI signaling pathway | 2 |
| 04666 | Fc gamma R-mediated phagocytosis | 11 |
| 04670 | Leukocyte transendothelial migration | 1 |
| 04062 | Chemokine signaling pathway | 2 |
| 04911 | Insulin secretion | 1 |
| 04910 | Insulin signaling pathway | 14 |
| 04922 | Glucagon signaling pathway | 15 |
| 04923 | Regulation of lipolysis in adipocytes | 1 |
| 04920 | Adipocytokine signaling pathway | 6 |
| 03320 | PPAR signaling pathway | 6 |
| 04912 | GnRH signaling pathway | 3 |
| 04913 | Ovarian steroidogenesis | 1 |
| 04915 | Estrogen signaling pathway | 5 |
| 04914 | Progesterone-mediated oocyte maturation | 6 |
| 04921 | Oxytocin signaling pathway | 7 |
| 04926 | Relaxin signaling pathway | 1 |
| 04935 | Growth hormone synthesis, secretion and action | 2 |
| 04918 | Thyroid hormone synthesis | 5 |
| 04919 | Thyroid hormone signaling pathway | 10 |
| 04928 | Parathyroid hormone synthesis, secretion and action | 2 |
| 04916 | Melanogenesis | 2 |
| 04924 | Renin secretion | 2 |
| 04614 | Renin-angiotensin system | 2 |
| 04925 | Aldosterone synthesis and secretion | 1 |
| 04260 | Cardiac muscle contraction | 9 |
| 04261 | Adrenergic signaling in cardiomyocytes | 7 |
| 04270 | Vascular smooth muscle contraction | 3 |
| 04970 | Salivary secretion | 2 |
| 04971 | Gastric acid secretion | 1 |
| 04972 | Pancreatic secretion | 5 |
| 04976 | Bile secretion | 5 |
| 04973 | Carbohydrate digestion and absorption | 3 |
| 04974 | Protein digestion and absorption | 3 |
| 04975 | Fat digestion and absorption | 4 |
| 04979 | Cholesterol metabolism | 4 |
| 04978 | Mineral absorption | 3 |
| 04962 | Vasopressin-regulated water reabsorption | 6 |
| 04960 | Aldosterone-regulated sodium reabsorption | 1 |
| 04961 | Endocrine and other factor-regulated calcium reabsorption | 4 |
| 04964 | Proximal tubule bicarbonate reclamation | 2 |
| 04966 | Collecting duct acid secretion | 8 |
| 04724 | Glutamatergic synapse | 5 |
| 04727 | GABAergic synapse | 8 |
| 04725 | Cholinergic synapse | 1 |
| 04728 | Dopaminergic synapse | 9 |
| 04726 | Serotonergic synapse | 2 |
| 04720 | Long-term potentiation | 4 |
| 04730 | Long-term depression | 2 |
| 04723 | Retrograde endocannabinoid signaling | 17 |
| 04721 | Synaptic vesicle cycle | 19 |
| 04722 | Neurotrophin signaling pathway | 6 |
| 04744 | Phototransduction | 2 |
| 04745 | Phototransduction - fly | 1 |
| 04740 | Olfactory transduction | 2 |
| 04742 | Taste transduction | 1 |
| 04750 | Inflammatory mediator regulation of TRP channels | 2 |
| 04360 | Axon guidance | 4 |
| 04361 | Axon regeneration | 9 |
| 04380 | Osteoclast differentiation | 2 |
| 04211 | Longevity regulating pathway | 12 |
| 04212 | Longevity regulating pathway - worm | 15 |
| 04213 | Longevity regulating pathway - multiple species | 12 |
| 04710 | Circadian rhythm | 7 |
| 04713 | Circadian entrainment | 2 |
| 04711 | Circadian rhythm - fly | 1 |
| 04712 | Circadian rhythm - plant | 15 |
| 04714 | Thermogenesis | 52 |
| 04626 | Plant-pathogen interaction | 21 |
| 05200 | Pathways in cancer | 22 |
| 05202 | Transcriptional misregulation in cancer | 10 |
| 05206 | MicroRNAs in cancer | 10 |
| 05205 | Proteoglycans in cancer | 8 |
| 05204 | Chemical carcinogenesis - DNA adducts | 2 |
| 05207 | Chemical carcinogenesis - receptor activation | 7 |
| 05208 | Chemical carcinogenesis - reactive oxygen species | 42 |
| 05203 | Viral carcinogenesis | 24 |
| 05230 | Central carbon metabolism in cancer | 9 |
| 05231 | Choline metabolism in cancer | 8 |
| 05235 | PD-L1 expression and PD-1 checkpoint pathway in cancer | 7 |
| 05210 | Colorectal cancer | 7 |
| 05212 | Pancreatic cancer | 7 |
| 05225 | Hepatocellular carcinoma | 10 |
| 05226 | Gastric cancer | 6 |
| 05214 | Glioma | 5 |
| 05216 | Thyroid cancer | 3 |
| 05221 | Acute myeloid leukemia | 2 |
| 05220 | Chronic myeloid leukemia | 3 |
| 05217 | Basal cell carcinoma | 2 |
| 05218 | Melanoma | 3 |
| 05211 | Renal cell carcinoma | 3 |
| 05215 | Prostate cancer | 6 |
| 05213 | Endometrial cancer | 5 |
| 05224 | Breast cancer | 5 |
| 05222 | Small cell lung cancer | 5 |
| 05223 | Non-small cell lung cancer | 3 |
| 05166 | Human T-cell leukemia virus 1 infection | 24 |
| 05170 | Human immunodeficiency virus 1 infection | 19 |
| 05161 | Hepatitis B | 5 |
| 05160 | Hepatitis C | 8 |
| 05171 | Coronavirus disease - COVID-19 | 76 |
| 05164 | Influenza A | 13 |
| 05162 | Measles | 8 |
| 05168 | Herpes simplex virus 1 infection | 15 |
| 05163 | Human cytomegalovirus infection | 8 |
| 05167 | Kaposi sarcoma-associated herpesvirus infection | 12 |
| 05169 | Epstein-Barr virus infection | 19 |
| 05165 | Human papillomavirus infection | 24 |
| 05110 | Vibrio cholerae infection | 12 |
| 05120 | Epithelial cell signaling in Helicobacter pylori infection | 11 |
| 05130 | Pathogenic Escherichia coli infection | 20 |
| 05132 | Salmonella infection | 26 |
| 05131 | Shigellosis | 31 |
| 05135 | Yersinia infection | 7 |
| 05133 | Pertussis | 2 |
| 05134 | Legionellosis | 11 |
| 05152 | Tuberculosis | 17 |
| 05100 | Bacterial invasion of epithelial cells | 7 |
| 05146 | Amoebiasis | 2 |
| 05145 | Toxoplasmosis | 4 |
| 05140 | Leishmaniasis | 2 |
| 05142 | Chagas disease | 4 |
| 05143 | African trypanosomiasis | 1 |
| 05322 | Systemic lupus erythematosus | 6 |
| 05323 | Rheumatoid arthritis | 10 |
| 05340 | Primary immunodeficiency | 1 |
| 05010 | Alzheimer disease | 78 |
| 05012 | Parkinson disease | 73 |
| 05014 | Amyotrophic lateral sclerosis | 92 |
| 05016 | Huntington disease | 78 |
| 05017 | Spinocerebellar ataxia | 38 |
| 05020 | Prion disease | 67 |
| 05022 | Pathways of neurodegeneration - multiple diseases | 92 |
| 05030 | Cocaine addiction | 1 |
| 05031 | Amphetamine addiction | 5 |
| 05032 | Morphine addiction | 2 |
| 05033 | Nicotine addiction | 1 |
| 05034 | Alcoholism | 13 |
| 05417 | Lipid and atherosclerosis | 13 |
| 05418 | Fluid shear stress and atherosclerosis | 9 |
| 05410 | Hypertrophic cardiomyopathy | 3 |
| 05415 | Diabetic cardiomyopathy | 44 |
| 05416 | Viral myocarditis | 3 |
| 04930 | Type II diabetes mellitus | 3 |
| 04940 | Type I diabetes mellitus | 2 |
| 04936 | Alcoholic liver disease | 12 |
| 04932 | Non-alcoholic fatty liver disease | 31 |
| 04931 | Insulin resistance | 12 |
| 04933 | AGE-RAGE signaling pathway in diabetic complications | 2 |
| 04934 | Cushing syndrome | 1 |
| 01502 | Vancomycin resistance | 1 |
| 01521 | EGFR tyrosine kinase inhibitor resistance | 5 |
| 01524 | Platinum drug resistance | 8 |
| 01523 | Antifolate resistance | 6 |
| 01522 | Endocrine resistance | 3 |
