# Supplementary material for: De novo transcriptome analysis and functional annotation of Silybum Marianum L. under drought stress with a focus on Silymarin synthesis and MAPK signaling pathways
Source: BMC Plant Biol. 2025 Aug 28;25:1150. doi: 10.1186/s12870-025-07272-5 (PMC12392515; doi:10.1186/s12870-025-07272-5)
Supplement: Supplementary file 3 — Supplementary Material 3 [file 12870_2025_7272_MOESM3_ESM.docx]

Sup3. KAAS protein families of Milk thistle genes

| **Protein families** | | |
| --- | --- | --- |
| **Metabolism** | | |
| **Enzymes** | Oxidoreductases | 3BETAHSDD, AASS, ACOX, acsF, ADH, ALDH, ALKBH5, AOC3, aroDE, BCKDHB, CAD, CAT, CBR1,  CCD8, CCR, CEQORH, CKX, COQ6, COX15, CRR1, crtZ, CRYZ, CYP, D2HGDH, DAO, DBR, DET2, DHAR, DHCR, DHFR-TS, DHODH, DHQ-SDH, DHRS12, DLD, DOPA, DOX, DUS, DVR, dxr, ETHE1, F3H, F6H, FAB2, fabI, FAD, FAH, FaQR, FAR, FDH, FDXR, FMO, frmA, FTH1, FTRC, G6PD, GA20ox, GAPA, GAPDH, gapN, gcpE, GLDC, GLT1,GLUD1_2,  GLYR, GPD1, gpx, GSR, HAO, HGD, hisD, HMGCR, HO, HPD, HPR1, HSD1, HY2, IDH, IFA38, ilvC, IMDH, IMPDH, IVD, JMJ30, KAR, KDM3, laccase, LDHD, LOX2S, LUT, maeB, MDH, MECR, metF, MFP2, MIOX, mmsA, msr, mtnD, nadB, NCED, NDC1,  ndh1, NOA1, NR, NXN, NYC1, OAR1, OGDH, OPR, P4HA, PAO, PCBER1, PCO, pdh, peroxidase, petH, PGD, PHYH, PIR, POR, PPOX, PRDX, PRXII, psbA, QSOX, RBOH, REF1, ribD, SC5DL, SDH, sir, SLD, SMO2, SOD, SQLE, SSADH, TER, thrA, TR1, trxB, TSTA3, TYRAAT, UGDH, VDE, VEP1, wrbA, YUCCA, ZDS, ZEP |
|  | Transferases | ABA, ACA, accA, ACLY, ADK, AGPAT3, AGXT, AHK, ALDH18A1, ALG, AMFR, ARAD, argAB, ARIH1, aroF, ATCYSC1, ATL6S, ATM, BAK1, BB, BIN2, BOI, BRAP, BRE1, BRI1, BSK, CALS, CARM1, CASD1, CBLL1, CDC2L, CDK, CERK, CESA, cfa, CHK, CHS, CISZOG, CMPK1, CNOT4, cobA, COMT, COQ, CPK, CRLS, crtB, CS, CSL, CSNK, CTR1, CTU1, cys, DCA, DGD, dgkA, DHDDS, DHFR-TS, DHPS, DIM1, DLAT, DLST, DNMT1, DPH5, EEF2KMT, EHMT, EIF2AK4, ELP3, EP300, fabF, FANCL, FDPS, FHY, FKGP, FOLK, FTSJ1, FUT13, GALK2, GALT, GAUT, GBE1, GGT1, GLCAK, glg, glmS, GLY, gmk, GMPP, GOLS, GOT1, GPAT, GPHN, GRC3, GST, GTK, GXM, HCT, HENMT1, HGSNAT, hisG, HPGT, HPT, IBA57, ICMT, ipk, IPMK, IPPK, ispD, ITPK4, KCS, kdsB, kdtA, LCLAT1, LPCAT1, LTN1, LYS, MAK, malQ, MAP3K3, MARCH6, MEKK, MENG, MET, MGAT, MGD, miaA, MKK, mmuM, MOCS, MPK, mraY, mtnK, MTOR, MUL1, NAA40, nadC, NAS, NAT, NCL1, ndk, NDUFAF5, NEK1, NMNAT, NMT, NOP, NRP, OGT, OSGEP, OXI1, OXSR, pabAB, PAP, PCYT2, PDPK1, PFK, pfp, PGK, PGTB1, PHOT, PHYLLO, PI4K, PIAS1, PIG, PIK, PIKFYVE, PIP5K, PK, PNKP, POL, POLRMT, POP2, PPIP5K, ppnK, PRKAA, PRMT1, PRPF, PTDSS2, purH, PYG, pyrH, QUA2, RABGGTA, RAY1, RBCMT, rbsK, RBX1, RCHY1, RDR, relA, REV1, RFWD2, RGLG, RGP, RHF, RIOK1, RIT1, rlmN, RMND5, RNF, RNGTT,  RNMT, rocD, RP, RPS6KB, RRT, rsm, SAMT, scrK, SELENOO, SETD3, SETMAR, SGT1, SHPRH, SIAH1, SIRT4, SIS3, SMARCA3, SMG1, SMT1, SMYD4, SNRK2, SPB1, speE, SPT, SQD2, SRPK3, STK, STT3, STUB1, SUS, SUVH1, SYVN1, TAF1, TAT, TCH4, Tdk, TGL4, TGS1, THG1, THI, thrA, tktA, TLK, tlyA, TOGT1, TPS, TRDMT1, TRIP12, TRM, trpD, TST, TUL1, UAP1, UBE, UBR7, Udk, UFL1, UGP, UGT73C, UHRF1, ULK2, VTC2, VTE3, WAXY, WNK, XBAT32, XEG11, XERICO, XK1, xylB, XYLT, YDA, ZAK, ZDHHC, ZNF598 |
|  | Hydrolases | abfA, ABHD17, ACER3, ACOT, ACY1, ADPRM, AFG, AGA, agu, ahcY, alkA, allC, amiE, AMPD, AMY, APF2, APTX, ASRGL1, ATAD2, ATG4, ATXN3, bgl, CARP, CEP, CHIB, clpP, CPL3, CSE, CTD, CTS, cysQ, DBR1, DCP, DDX, DEP1, DESI, DHX, DICER1, DIS3, DNM1L, DNPEP, DPP, DPYS, DRG, dtdA, DUSP12, EBM, EGY, EIF4A3, EME1, ENDO, ENPP1, ENTPD1, ERI2, ESP1, EYA1, FAHD1, FAN1, FAT, FBP, FEN1, FHY, FIG4, frmB, ftsH, FUCA, GBA2, GCH1, GDE1, GEN1, GGH, GGT1, gloB, GN1, HDAC1, HEXA, HIBCH, hisIE, HM13, HPSE, HSPA5, HTRA2, ICT1  IDE, IMP2, IMPL2, INV, ISA, lacZ, LCAT3, lepB, LGMN, LIPA, LOG, lon, LPIN, LYPLA2, MAN, map, MBTPS1, MCA1, MGLL, MINDY, MIPEP, mnmE, MOGS, MPG, MPPE1, MTMR1, MTN, MUS81, NAALAD, NAGLU, NGLY1, NMA111, NOTUM, NPEPPS, NSF, NTAN1, NTPCR, NUD, OGG1, orn, otsB, OUT, PAR, PCME, PDP, pep, PFKFB3, PGAP1, PGLS, PGP, PHOSPHO2, PIGL, pip, PITRM1, PLA2G, plc, PLCD, PLD1, PMPC, PNKP, POP4, PP2C, ppa, PPM, PPP, prc, PRCP, PRDX6, PREP, PRORP, PSM, PTC, PTEN, pur, PYRP2, RAD, RCE1, REXO4, RHBDD1, RHOT1, RIA1, rib, RNASEH2A, RNGTT, RPAP2, RRP6, rsgA, SACM1L, SAL, SAR1, SCPL, SEC11, SENP1, SEY1, SIW14, SKI2, SNRNP200, SPCS2, SPP, SRP54, STAMBP, SUPV, surE, TAD1, tadA, tag, TGL4, TPP2, TPS, TREH, TTHL, TYSND1, UBLCP1, UFSP2, ULP1C, UPF1, URA4, URE, USB1, USP, USP15, VTC4, WRNexo, wzb, XCP, YGK1, ylbA, YME1, ZUP1 |
|  | Lyases | ACO, ACS1, ADCL, ADT, ALDO, ALKBH1, AOS, APEX2, aro, ATCYSC1, CARKD, CER1, cynT, DDC, DEP1, DJ1D, DPL1, ECH2, ENO, gadB, GAMMACA, GERD, GGCT, GLO1, gmd, HACD, HACL1, hem, HIS7, HMGCL, HPL, ilv, ispS, leuC, ltaE, MDL3, menB, metC, MFP2, MLYCD, moaA, MVD, NES1, NIT4, NTH, OGG1, paaF, PAL, PCBD, PDC, pdx, pel, phr, PHYLLO, PISD, POLL, ppc, purB, rbc, RGL4, RHM, ribBA, RNASET2, SDC, SGR, sirB, speD, sufS, thiC, thrC, trp, TYW1, UXS1 |
|  | Isomerases | AOC, ASCC3, BTAF1, CHD, CPI1, crtISO, DKC1, ECI1, FIGNL1, FKBP, FPR3, GAE, galM, GPI, gpm, HFM1, hisA, INO, KATNA1, lcyE, LUP4, maiA, manA, menF, mtnA, PDIA, PEO1, pgm, PGM3, PHYLLO, PIF1, PIN4, PPI, PPIL4, PTGES2, RAD, RECQL, RGP, rpe, rpiA, RUVBL2, SHPRH, SMARC, SRR, TFIIF2, TOP1, TPI, tru, VTE1, xylA |
|  | Ligases | 4CL, AAE, AASDH, ACACA, acc, ACSL, ACSS1, argG, CARS, chlH, DARS1, DHFS, DPH6, FARS, fhs, FPGS, gat, glnA, guaA, HARS, HLCS, IARS, JAR1, KARS, LARS, LIG1, LSC, MARS, PARS, pncB, PPCS, purM, pyrG, SARS, TARS, UBA3, UBE1, UBLE1B, VARS, WARS, YARS |
|  | Translocases | ND5, NDUF, ndh, petC, UQCRFS1, COX1, PMA1, ATPF1B, ATPe, AVP, CYB561, copA, ATP2C, zntA, arsA, secA, DRS2, ABCB1 |
| **Protein Kinases** | Serine/threonine kinases: AGC group | PDPK1, RPS6KB, STK38, OXI1, PHOT |
|  | Serine/threonine kinases: CAMK group | PRKAA, SNRK2, CPK |
|  | Serine/threonine kinases: CK1 group | CSNK1E |
|  | Serine/threonine kinases: CMGC group | CDC2L, CDK, MPK6, BIN2, PRPF4B, MAK, SRPK3 |
|  | Serine/threonine kinases: STE group | MKK, OXSR, STK, MAP3K3, YDA |
|  | Serine/threonine kinases: TKL group | BRI1, BSK, ZAK, CTR1 |
|  | Serine/threonine kinases: Other | CAMKK1, CSNK2A, NEK1, EIF2AK4, SCYL1, TLK, ULK2, PIK3R4, WNK, ADCK, STK19, ATM, MTOR, SMG1, RIOK1, TAF1, DCAF1 |
|  | Receptor serine/threonine kinases (RSTK): TKL group | - |
|  | Receptor tyrosine kinases (RTK) | - |
|  | Non-receptor tyrosine kinases | - |
|  | Histidine kinases | - |
| **Protein Phosphatases and Associated Proteins** | Protein serine/threonine phosphatases | PPP1, DDX31, GPATCH2, CD2BP2, CENPE, CLCN7, GRXCR1, ahcY, KDM5, RPL5, NOM1, pfkA, RBM26, SMARCB1, SRSF10, TRA2, VPS54, WDR81, WNK, YLPM1, ZSWIM3, PPP2, TIPRL, NXN, PPP3R, CALM, CABIN1, SMEK, HSP90A, HSPA1s, PDP |
|  | Protein tyrosine phosphatases (PTPs) | - |
|  | HAD phosphatases | - |
| **Peptidases and Inhibitors** | Aspartic peptidases | Phytepsin, APF2, HM13, SPPL2B |
|  | Cysteine peptidases | CEP, CTSF, CTSH, XCP, LGMN, MCA1, USP, GGH, guaA, glmS, SENP1, ESP1, ATG4, DJ1D, OTUB1, UFSP2, OTUD5, ATXN3, OTU1, DESI |
|  | Glutamic peptidases | RCE1 |
|  | Metallo peptidases | pepN, NPEPPS, TAF2, MIPEP, GP63, IDE, PITRM1, PMPC, DNPEP, ACY1, allC, map, pepP, NAALAD, ftsH, AFG3, YME1, EGY, STAMBP |
|  | Mixed peptidases | - |
|  | Serine peptidases | HTRA2, TYSND1, NMA111, MBTPS1, TPP2, PREP, DPP4, ABHD17, SCPL, clpP, lon, lepB, SEC11, IMP2, PRCP, pip, MGLL, PPME1, prc, sppA, RHBDD1 |
|  | Threonine peptidases | PSM, ASRGL1, GGT1, |
|  | Peptidases of unknown catalytic type | - |
|  | Peptidase inhibitors | - |
| **Glycosyltransferases** | N-Glycan biosynthesis | ALG |
|  | O-Glycan biosynthesis | OGT, SGT, GALT2S, HPGT |
|  | GPI-anchor biosynthesis | PIGB |
|  | Glycolipid biosynthesis | MGD, SQD2 |
|  | Glycan extension | MGAT, XYLT, FUT13, XEG113, DGD |
|  | Terminal extension | - |
|  | Polysaccharide | glgA, WAXY, CESA, CALS, GALT29A, RGP, GAUT, CSL, GLCAT14, RAY1, ARAD, RRT |
|  | Others | IAGLU, UGT, TPS, SUS |
| **Lipopolysaccharide Biosynthesis Proteins** | Lipid A | kdtA, kdsB, kdsD |
|  | Core region | - |
|  | O-antigen repeat unit | - |
| **Peptidoglycan Biosynthesis and Degradation Proteins** | - | - |
| **Lipid Biosynthesis Proteins** | Fatty acid synthase | Fab, HSD17B12, HACD, TER, FAT, PPT, ACOT |
|  | Desaturase | FAB2, FAD |
|  | Elongase | - |
|  | Polyketide synthase | - |
|  | Acyl-CoA synthetase | ACSS1, AAE7, ACSL, AASDH |
|  | Phospholipid acyltransferase | GPAT, LCLAT1, AGPAT3 |
| **Prenyltransferases** | Terpene biosynthesis | FDPS, SPS, DHDDS, crtB |
|  | Compound prenylation | miaA, HPT, COQ2 |
|  | Protein prenylation | PGTB1, RABGGT, CHM, PTAR1 |
| **Amino Acid Related Enzymes** | Aminoacyl-tRNA synthetase | YARS, WARS, CARS, MARS, IARS, VARS, LARS, KARS, DARS1, HARS, SARS, PARS, TARS, FARS |
|  | Aminotransferase (transaminase) | GOT, GGAT, TAT, ACS, bioF, SPT, rocD, ilvE, AGXT |
| **Cytochrome P450** | Cytochrome P450, plant type | CYP71A16, CYP73A, AOS, CYP75B1, CYP76C, CYP77A, CYP78A, CYP79, CYP81D, CYP82C4, CYP84A, CYP85A2, CYP86A4S, CYP89A, CYP90C1, LUT, CYP98A, CYP707A, CYP714A1, CYP735A |
| **Photosynthesis Proteins** | Photosystem and electron transport system | psb, psa |
|  | Antenna proteins | LHCA, LHCB |
|  | Anoxygenic photosystem | - |
| **genetic information processing** | | |
| **Transcription Factors** | Eukaryotic type | TGA, GBF, HY5, ABF, VIP1, CCNDBP1, PIF3, MYC2, ATRX, GTF3A, RFA1, CNOT4, DNMT1, CNBP, HD-ZIP, HSFF, MYBP, MADS-box, TBP, NFY, EP300, AP2, EREBP, ERF1, WRKY,‌ CAMTA, EIN3 |
| **Transcription Machinery** | Eukaryotic type | RPB, RPABC, NRPD, NRPE, TFIIA1, TBP, TAF, TFIIH, CCNH, MED, CDK8, ENY, USP22, SMARC, ACTL6A, PCF, CPSF, PPP, SYMPK, XRN2, RAI1, RTT103, SETX, CTR9, CCNT, LARP7, BTAF1, DR1, CPL3, RPAP, GPN, RPC, RPABC, GTF3A, TBP, GTF3C, SNAPC, RPA, TBP, RRN3, SMARCA5 |
| **Messenger RNA Biogenesis** | Eukaryotic type | RNMT, RNGTT, NCBP, NUDT, CPSF, PCF, WDR, PPP1C, CSTF, SYMPK, SNRPB, SNRPD3, CSTF2, METTL14, CBLL1, PAP, BARD1, RBM26, ALKBH5, RBM8A, THOC4, DDX39B, EIF4A3, ACIN1, SRRM1, THOC, PCID2, ENY2, SAC3, TPR, RAE1, NUP, GLE, RAN, VCP, XPO1, EIF, CYFIP, DDX3X, PABPC, SKI2, DENR, WIBG, HNRNPA1, MSI, HBS1, PABPN1, DDX19, ERF, UPF, PPP2, SMG7, MYO5, EXD1, FUBP, EEF1, CYFIP, PCBP3, ELF2C, UPF1, DDX3X, LSM14, SMG7, HNRNPA1, LARP1, DCP, LSM, MYO, TUBA, CNOT, EDC, UPF, YTHDF, TIA1, PABPC, EIF, RACK, DCP, EDC, LSM, XRN2, RRP, DIS, MPHOSPH6, PAPD5, SKI, CNOT, CAF, PARN, DHX |
| **Spliceosome** | Common components | NCBP, HNRNPA1, SRSF, TRA, RBM23 |
|  | Complex A | SNRP, DDX, PRPF, RBM, TCERG1, SNRP, SF3, DDX, U2AF, RBM, SMNDC, SR140, ZRSR, CDC2L, RBM5, SF, TLS |
|  | Complex B | SNRP, DDX, PRPF, RBM, TCERG, SF3, U2AF, SMNDC1, SR140, ZRSR, LSM2, SART3, EFTUD2, SNRNP200, CD2BP2, PRPF38, CWC22, DHX16, PRPF19, CDC5L, PLRG1, CWC15, HSPA1s, SYF2, CCDC12, RBM22, IK, SMU1, UBL5 |
|  | Complex C | SNRP, LSM2, SF3, DDX, EFTUD2, PRPF38, CWC22, DHX16, TFIP11, PRPF19, CDC5L, CWC15, PLRG1, HSPA1s, SYF2, CCDC12, RBM22, ACIN1, RBM8A, THOC, DHX, PPI, HNRNP, DGCR14, FRA10AC1, NOSIP, PABPC, TFIP11, ZCCHC8, CACTIN |
|  | Other splicing related proteins | GEMIN2, STRAP, COIL, SMNDC1, USB, TGS, SNRNP25, ZCRB1, RNPC3, RBM48, ARMC7, CLASRP, AKAP17, SRRM1, THOC, DDX, RBM, QKI, SERBP1, TIA1, YTHDC1, AAR2, FUBP, PPP, PRPF, PTBP2, CCNL, DBR1 |
|  | Spliceosomal RNAs | - |
| **Ribosome** | Ribosomal proteins | RP-S, RP-L, DAP3, MRPS33, PSRP3 |
|  | Ribosomal RNAs | - |
| **Ribosome Biogenesis** | Eukaryotic type | - |
| **Transfer RNA Biogenesis** | Eukaryotic type | POP4, PRORP, rnz, RNASET2, MATK, RAN, XPOT, XPO, EEF1A, SCYL1, PARS, IARS, LARS, MARS, KARS, DARS1, TARS, SARS, CARS, VARS, HARS, FARS, YARS, WARS, dtdA, TAD, DUS, TRMT2A, NCL1, TRM5, METTL6, ALKBH8, TRMT, TRM, TRDMT, TRMO, TYW1, tru, CTU, MOCS, OSGEP, TPRKB, ELP, KTI12, miaA, mnmE, gidA, RIT1, THG1, PAPD5, XRN2, cysQ |
| **Translation Factors** | Eukaryotic type | EIF1A, EIF, inf, ABCF1, TIF31, DHX29, TBL2, DPH, EEF2KMT |
| **Chaperones and Folding Catalysts** | Heat shock proteins | - |
|  | Other chaperones and cochaperones | - |
|  | Intramolecular chaperones | - |
|  | Protein folding catalysts | - |
| **Membrane Trafficking** | Exocytosis | PDPK1, PLCD, PLD1, PIP5K, STK24, ndk, RAB8A, RAB3GAP1, CALM, STXBP1, PEX5, CHM, RABGGTA, DMXL, RAC1, EXOC, SCAMP |
|  | SNARE | STX, VTI1, NSPN, GOSR1, SYP, BET, USE, SNAP25, ATVAMP72, VAMP7, SEC22, STXBP5, STXBP1, ZDHHC, NSF, NAPA, VCP, NSFL1C |
|  | Endocytosis | CLTC, DNM1L, AP2, EPN, EPS15, HSPA1s, PIP5K, CBLL1, CANX, ANKRD13, AAGAB, DNM1L, ARF1, RAC1, DNM1L, RAC1, PIK3C3, PIKFYVE, PLCD, RABGEF1, PIK3, VPS45, ZDHHC1, ARF, ACAP, SMAP, ELMOD, PI4KA, EFR3 |
|  | Endosome - Lysosome transport | RAB7A, VPS, RNF115, PIK3C3, PSMA7, TBC1D15, CHM, RABGGT, NAA, VPS, CHMP, PDCD6IP, DMXL, SKP1, AP3M, MON1, RMC1, STAMBP, CCDC22 |
|  | Protein recycling | RAB11A, EVI5, EHD1, TBC1D13, RABGEF1, RAD51, ACAP, VPS, CAPZB, EIPR1 |
|  | Endosome - Golgi transport | TMF1, VPS13A, ARF1, ARFRP1, SMAP, ARFGEF, GBF1, COG, VPS, TRAPPC, AP, PLD1, PI4KA, PIP5K, PIKFYVE, TMEM165, SERINC1 |
|  | Endoplasmic reticulum (ER) - Golgi transport | SAR1, SEC, TRAPPC6, TMED10, PDIA, RAB1A, RABAC1, TBC1D22, MAN1, ERGIC3, CNIH, COP, NBAS, RINT1, arsA, RIC1, RGP1, TBC1D22, OSBP, EMC, PGAP, MPPE1, ARL6IP5, TMEM33, RTN3, PIEZO1, DYM, SEY1 |
|  | Autophagy | ATG, ULK2, ATG13, PIK3, BECN, WIPI1, GABARAP, MAN2C1, DNPEP, CLEC16A, NBR1, AMBRA1, HK, NBR1, PEX1, WDFY3, VCP, STUB1, ALDO, GOT1, GAPDH, PK, UBQLN, HSP, ST13, EEF1A, MTOR, RAB, PRKAA, PPP2C, WDR45 |
|  | Others | RAC1, MYO5, ARPC, ACTR, SH3YL1, PFN, CFL, RAB18 |
| **Ubiquitin System** | Ubiquitins and ubiquitin-like proteins | - |
|  | Ubiquitin-activating enzymes (E1) | - |
|  | Ubiquitin-conjugating enzymes (E2) | - |
|  | Ubiquitin ligases (E3) | RBX1, CUL1, SKP, FBXL2, COI, EBF1, TIR1, GID2, ZTL, RBX1, ABTB1, SPOP, NPR1, RBX1, CUL4, DDB, ERCC8, DET1, RFWD2, DCAF1, PHIP, WDR26, ROC1, CBF3D, ANAPC5, CDC23 |
|  | Deubiquitinating enzyme (DUB) | - |
| **Proteasome** | Eukaryotic proteasome | PSMC, PSMD |
| **DNA Replication Proteins** | Eukaryotic type | GAR1, DKC1, SNRP |
| **Chromosome and Associated Proteins** | - | - |
| **DNA Repair and Recombination Proteins** | Eukaryotic type | POLD, RAD23, RBX1, CUL4, DDB, RPB, RPABC, ERCC8 |
| **Mitochondrial Biogenesis** | Mitochondrial DNA transcription, translation, and replication factors | ND, CYTB, COX, POLRMT, inf, tsf, tuf, fusA, MTERFD, POP4, SUPV3L1, SLC25A4S, RP-S6, era, YARS, LARS, gat, MTG1, NOA1, PEO1, PIF1, ssb, ATAD3A |
|  | Mitochondrial protein import machinery | DNAJA2, RHOT1, HSPA1s, TOM40, SAM50, VDAC2, MICOS10, IMMT, TIM21, MCU, MICU1, LETM1, IMP2, MPC1, TIM44, dnaK, GRPE, PMPC, MIPEP, groEL, dnaJ, TAM41, ETHE1 |
|  | Mitochondrial quality control factors | fusA, EP300, ISCU, LYRM4, FDXR, dnaK, IBA57, NFU1, BOLA1, PRKAA, NCA2, NDUFAF, RTN4IP1, CMC1, COA6, COX, yidC, SURF1, SCO1, LRPPRC, OPA3, DNM1L, PHB, DNM1L, MYO5, RHOT1, IMMT, DAP3, ATG, BECN, GABARAP, WIPI1, CLEC16A, AMBRA1, ULK2, HSP90A, PRKAA, NBR1 |
| **Signaling and cellular processes** | | |
| **Transporters** | ABC transporters, eukaryotic type | ABCA, ABCB, ABCC10, ABCG2 |
|  | ABC transporters, prokaryotic type | STAR1 |
|  | Solute carrier family (SLC) | - |
|  | Major facilitator superfamily (MFS) | STP, SLC2A13, ERD6, MFS, NRT, MFSD5 |
|  | Phosphotransferase system (PTS) | - |
|  | Other transporters | PIP, TIP |
| **Secretion System** | Type I secretion system | - |
|  | Type II secretion system | - |
|  | Type III secretion system | - |
|  | Type IV secretion system | - |
|  | Type V secretion system | - |
|  | Type VI secretion system | - |
|  | Chaperone-usher system | - |
|  | Extracellular nucleation-precipitation pathway | - |
|  | Sec (secretion) system | secA, SRP54, ftsY, yidC, SEC, SRP54, SEC31 |
|  | Twin-arginine translocation (Tat) system | tatC |
| **Cilium and Associated Proteins** | Primary cilia and associated proteins | CCT, NEK1, TUB, MAK |
|  | Motile cilia and associated proteins | DYNLL, RUVBL2 |
|  | Other cilia and associated proteins | ELMOD, GRXCR1 |
| **Cytoskeleton Proteins** | Eukaryotic cytoskeleton proteins | - |
| **Exosome** | Exosomal proteins | ABCB1, ABCG2, ACAP, ACAT, ACLY, ACSL, ACTR, ACY1, adk, ALDO, AP2A, APOD, APRT, ARF1, argG, ARHGDI, ATPeV, CALM, CAND1, CAPZB, CCT, CFL, CHMP, CLTC, COP, CTN, CUL4, DDC, DLD, dnaK, DNM1L, DPP4, DPYS, EEF, EHD1, EIF, ENO, FBP, FEN1, FTH, FUCA, G6PD, GAPDH, GBE1, GDI1, glnA, GLUD1, GNB, GOT1, GPI, groEL, H2, H3, hemB, HPD, HSP90A, HSPA1s, IMPDH, LSC2, MOGS, NAGLU, PDCD6IP, PDIA, PFN, PGK, PGRMC1, PHB, PK, PLS1, PPP2C, PRDX2, PSMF1, purH, RAB, RAB3GAP1, RAC, RACK, RAN, REEP5, RP-S27Ae, SELENBP1, SGTA, SLC25A3, STX1B, TM9SF2, TPI, TUB, TUBGCP2, tuf, UBE1, USP5, VAMP7, VCP, VPS, YWHAE, |
|  | Exosomal RNA | - |
| **Prokaryotic Defense System** | CRISPR-Cas system | - |
|  | Restriction and modification system (R-M system) | DNMT1 |
|  | Toxin-antitoxin system (TA system) | ftsZ |
|  | DNA phosphothiolation system | - |
| **G Protein-Coupled Receptors** | Rhodopsin family | - |
|  | Secretin receptor family | - |
|  | Adhesion receptor family | - |
|  | Metabotropic glutamate receptor family | - |
|  | Frizzled / Smoothened family | - |
|  | Others | GPR107, MLO, GCR1 |
| **Pattern Recognition Receptors** | Membrane-bound pattern recognition receptors | - |
|  | Cytoplasmic pattern recognition receptors | NLRC3 |
| **Ion Channels** | Ligand-gated channels | GRIP |
|  | Voltage-gated cation channels | TPC1, CNGC |
|  | Chloride channels | CLCN7, ANO10, VDAC2 |
|  | Other channels | PIEZO1 |
| **GTP-Binding Proteins** | Heterotrimeric G-proteins | GNAT3, GNB1 |
|  | Small (monomeric) G-proteins | RAC1, RHOT1, RAB1A, RAB7A, RAB8A, RAB11A, RAB18, RAN, ARF1, ARL2, ARFRP1, SAR1 |
| **CD Molecules** | Proteins | CD |
|  | Carbohydrates | - |
| **Glycosaminoglycan Binding Proteins** | Heparan sulfate / Heparin | HPSE, CTSB |
|  | Hyaluronan | C1QBP |
| **Glycosylphosphatidylinositol (GPI)-anchored Proteins** | Enzymes | ENPP1, TREH, GN1 |
|  | Receptors | - |
|  | Antigens | GP63 |
|  | Others | - |
| **Domain-containing proteins not elsewhere classified** | Src homology (SH) domain-containing proteins | - |
|  | Pleckstrin homology (PH) domain-containing proteins | - |
|  | Phosphotyrosine binding (PTB) domain-containing proteins | - |
|  | EF-hand domain-containing proteins | - |
|  | EVH1 / WASP homology (WH) domain-containing proteins | - |
|  | PDZ domain-containing proteins | - |
|  | Phox homology (PX) domain-containing proteins | - |
|  | Zinc finger domain-containing proteins | SMYD4 |
|  | LIM domain-containing proteins | - |
|  | Laminin domain-containing proteins | - |
|  | Fibronectin (FN) domain-containing proteins | - |
|  | C1q domain-containing proteins | - |
|  | Thrombospondin domain-containing proteins | - |
|  | EGF-like domain-containing proteins | - |
|  | Von Willebrand domain-containing proteins | - |
|  | WD40 repeat (WDR) domain-containing proteins | WDR |
|  | Other domain-containing proteins | TUB, IBTK, ARMC6, THADA, TTC27, RBM42 |
